# Magnitude and decline of pesticide co‐formulant residues in vegetables and fruits: results from field trials compared to estimated values

**DOI:** 10.1002/ps.6128

**Published:** 2020-10-26

**Authors:** Marianne E Balmer, Daniel Janser, Ulrich Schaller, Jürgen Krauss, H Christoph Geiser, Thomas Poiger

**Affiliations:** ^1^ Agroscope, Plant Protection Chemistry Wädenswil Switzerland; ^2^ Agroscope, Vegetable‐Production Extension Wädenswil Switzerland; ^3^ Federal Food Safety and Veterinary Office (FSVO) Toxicology and Biology Bern Switzerland

**Keywords:** pesticide, co‐formulant, residues in food, consumer exposure, plant protection product

## Abstract

**BACKGROUND:**

The application of plant protection products (PPPs) leads to the formation of residues in treated crops. Even though PPPs contain considerable amounts of co‐formulants, regulation and monitoring of residues normally focus on the active substances (a.s.) only. For our study we selected four commonly used co‐formulants (three anionic surfactants and one organic solvent) and investigated the formation and decline of residues in vegetables and apples under field conditions. The aims were to characterize the behavior of co‐formulant residues on crops and to provide a basis for future investigations on consumer exposure.

**RESULTS:**

The development of robust and sensitive analytical methods allowed the quantification of residues in the low μg/kg‐level. After treatment with PPPs, co‐formulants were detected up to approximately 10 mg kg^–1^ in vegetables. In general, these residues declined fast with half‐lives of a few days. Wash‐off and volatilization were identified as important removal processes for anionic surfactants and the organic solvent, respectively. However, in specific crops (parsley and celery), organic solvent residues were still considerable (≈2 mg kg^–1^) 2 weeks after treatment. We further demonstrate that it is feasible to estimate co‐formulant residues using publicly available data on pesticide a.s.

**CONCLUSION:**

To date no information on co‐formulant residues in food is available. The findings from our field trials, as well as the presented approach for the prediction of residues, provide key elements for future consideration of consumer exposure to PPP co‐formulants. © 2020 The Authors. *Pest Management Science* published by John Wiley & Sons Ltd on behalf of Society of Chemical Industry.

## Graphical abstract

We compare our field data on residues of selected pesticide co‐formulants to predicted values in an effort to provide a basis for future considerations on consumer exposure to these substances.

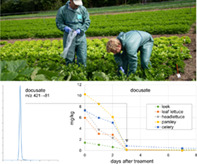

## 1 INTRODUCTION

Plant protection products (PPPs) consist of one or more active substances (a.s.), possibly a synergist or a safener, and (potentially numerous different) co‐formulants. Application of PPPs in the field inevitably leads to formation of residues on the treated crops. The European regulation concerning the placing of PPPs on the market^1^ defines residues as one or more substances present in or on plants or plant products, edible animal products, drinking water or elsewhere in the environment resulting from the use of a PPP, including their metabolites, breakdown or reaction products. In practice, the risk assessment and the regulation of residues are limited to the a.s. and selected metabolites, for which maximum residue levels in or on food are established.^2^ To date, residues of co‐formulants of PPPs are not considered and, consequently, very little information is available concerning their nature and magnitude.

While the contents of active substances, synergists, and safeners are specified on the packaging of every PPP, identity and content of co‐formulants are usually not publicly disclosed. This may be a further reason why information on occurrence and behavior of co‐formulants in the environment and in/on plants is scarce. Although several authors stated that co‐formulants may occur in the environment and can influence the (eco‐)toxicity of PPPs,^3^, ^4^, ^5^, ^6^, ^7^, ^8^, ^9^ to our knowledge there is no published data available to date, on potential or actual residues of co‐formulants in or on food.

In a previous study, we provided a quantitative overview on formulation types and content of co‐formulants in PPPs on the Swiss market, based on PPP compositions submitted for regulatory purposes to the responsible authority and on PPP sales figures.^10^ This evaluation revealed the five most relevant formulation types (in terms of mass sold) being soluble concentrates (SL), water dispersible granules (WG), emulsifiable concentrates (EC), suspension concentrates (SC), and wettable powders (WP). Overall, in these formulations the a.s. accounted for roughly 50% of the total mass, water for about 30%, and co‐formulants (including carrier materials) for the rest. On a mass basis, organic solvents were identified as the most important class of co‐formulants (10%), followed by surfactants (8%), with about two‐thirds anionic and one‐third non‐ionic surfactants. Other components were of minor importance.

In the present study, we intended to characterize the formation and decline of co‐formulant residues on vegetables and fruits under agricultural practice, and to provide a basis for future considerations of potential consumer exposure to these chemicals. Therefore, we conducted field residue trials with six different vegetable crops, as well as with apples. The vegetables were selected to cover a wide range of plant habitus: leek (*Allium porrum*), celery (*Apium graveolens var. dulce*), rondini (a ball‐shaped, edible peel variety of *Cucurbita pepo*), parsley (*Petroselinum crispum convar. crispum*), head lettuce (*Lactuca sativa var. capitata*), and oak leaf lettuce (*Lactuca sativa var. crispa*). Co‐formulants were selected based on the following criteria: (i) representativeness for an important group of co‐formulants; (ii) feasibility of a sensitive and robust analytical method; (iii) availability of reference compounds; (iv) commercial availability of PPPs with significant content of the respective co‐formulant.

Organic solvents are most important in EC formulations, with an average content of about 70%. Overall, PPPs sold in Switzerland in 2015 contained an estimated 360 t of organic solvents.^10^ From this co‐formulant class, *N,N*‐dimethyldecanamide (DMDA) was selected for this study even though total use in PPPs was comparably small (9 t/year). The main reasons for selecting DMDA were its moderate volatility that potentially leads to higher residues (when compared to other, more volatile solvents), as well as the fact that it is a single substance (and not a mixture) which greatly facilitates the analysis of residues.

**Figure 1:**
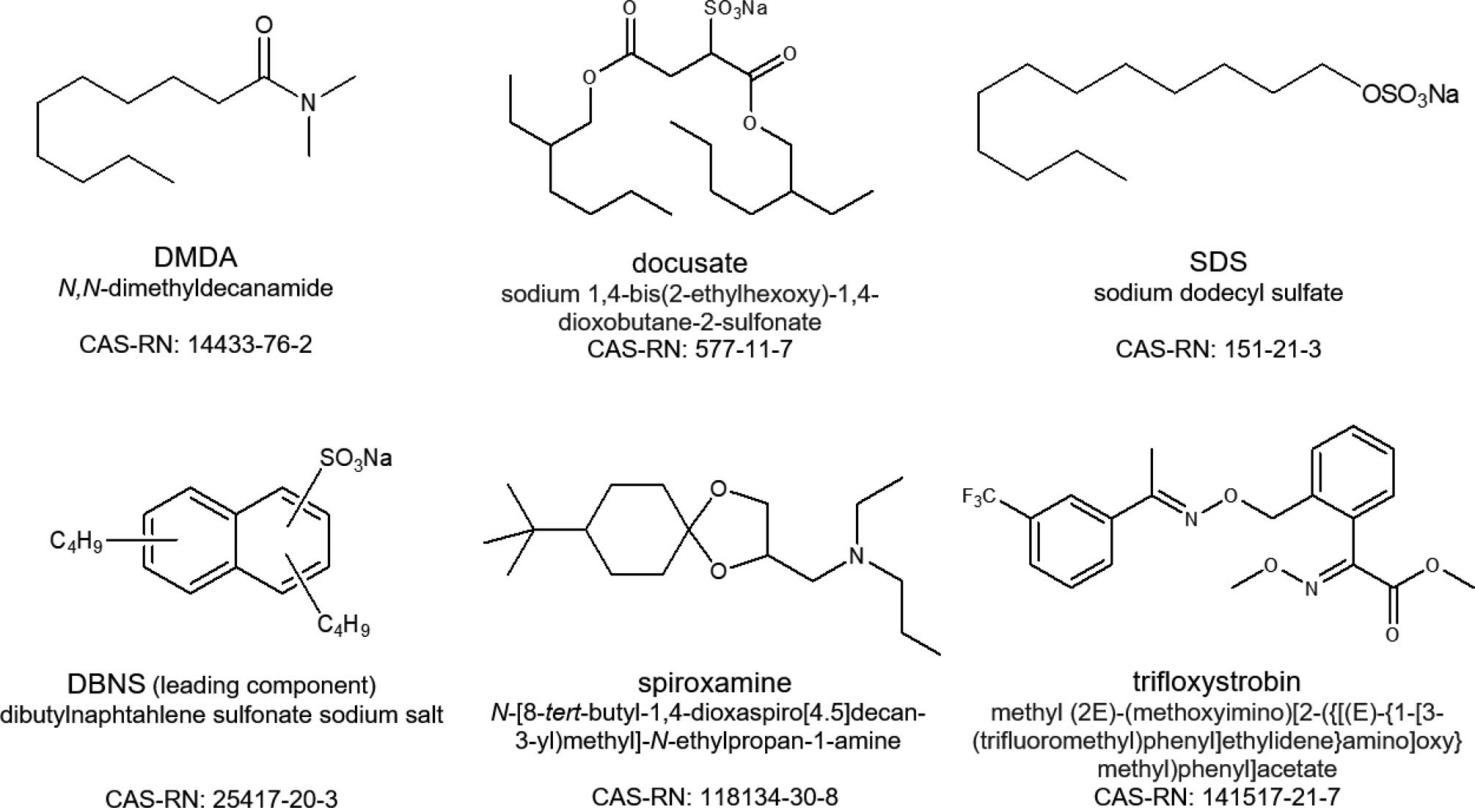
Chemical structures, common names, IUPAC names, and CAS‐RN of co‐formulants and active substances investigated in residue field trials.

The total amount of anionic surfactants contained in PPPs sold in 2015 in Switzerland was estimated at 190 t. Despite its comparatively minor use (1 ton in 2015, as estimated in^10^) we selected di‐2‐ethylhexyl sulfosuccinate (docusate) as a representative, because (as for DMDA) it is a single substance while many other anionic surfactants consist of a mixture of homologs and isomers which is often not well defined. We additionally monitored the residues of two other anionic surfactants that were present in the PPPs used in the field trials: sodium dodecyl sulfate (SDS) and DBNS, a surfactant that consists of a mixture of butylnaphthalene sulfonates with the lead component dibutylnaphthalene sulfonate. PPPs sold in Switzerland in 2015 contained an estimated total of roughly 0.14 and 0.25 tons SDS and DBNS, respectively.^10^ Further, spiroxamine and trifloxystrobin, the active substances in two of the applied PPPs, were also monitored for comparison. For chemical structures of all investigated substances see Fig. 1.

It should be noted that the use as co‐formulants in PPPs accounts only for a minor portion of the overall usage of these substances. Their uses in consumer products are manifold, including washing and cleaning agents and personal care products (compare e.g., ECHA registration dossiers^11^).

Three commercially available PPPs, containing one or two of the co‐formulants specified above, were applied in the field trials (Table 1), and the formation and decline of co‐formulant residues were monitored. We then applied an approach to estimate co‐formulant residues based on publicly available data for pesticide a.s., with the intent to evaluate the suitability of such generic estimation methods for exposure assessment within a regulatory framework.

**Table 1:** Plant protection products applied in field trials: content of active substances and relevant co‐formulants and application rates

PPP trade name	Formulation type	Application rate (PPP)	Amount of water	Common name	Function	Content in (PPP)	Application rate	Monitored
		(kg ha^–1^)	(L ha^–1^)			(g kg^–1^)	(g ha^–1^)	
**Trial in vegetables**								
Armicarb	SP	3	800	KHCO_3_	fungicide	(850)	(2550)	no
				docusate	surfactant	88	263	yes
				SDS	surfactant	62	168	yes
Flint	WG	0.4	800	trifloxystrobin	fungicide	514	206	yes
				DBNS	surfactant	47	19	yes
Input	EC	1.25	800	spiroxamine	fungicide	300	375	yes
				prothioconazole	fungicide	(160)	(200)	no
				DMDA	solvent	396	495	yes
**Trial in apples**								
Armicarb	SP	4.8	400	KHCO_3_	fungicide	(850)	(4080)	no
				docusate	surfactant	72	346	yes
				SDS	surfactant	54	257	yes
Flint	WG	0.24	400	trifloxystrobin	fungicide	502	120	yes
				DBNS	surfactant	47	11	yes

Reported contents of active substances (fungicides) and co‐formulants (surfactants or solvents) are based on measurements in the plant protection product. For potassium hydrogen carbonate (KHCO_3_) and prothioconazole, the declared content is reported (in brackets). Note: different batches of Armicarb were used in the vegetable and apple trial respectively, resulting in slightly varying docusate and SDS contents.

## 2 MATERIAL AND METHODS

### 2.1 Field trials and sampling procedure

Field trials in vegetables were conducted in Wädenswil, Switzerland (47°13′17”N/8°40′38″E, altitude, 485 m, loamy silt soil, pH 7.1). The field was divided into 36 plots (1.5 m x 7 m each). Leek (variety: Belton F1), rondini (Eight Ball), celery (Rumba), parsley (Katinka, crisp‐leafed variety), head lettuce (Analotta F1), and oak leaf lettuce (Kisheri F1) were each planted on six plots in a row. Planting dates (between June 03 and July 23, 2019) were chosen to achieve harvest stage of all crops at approximately the same time. The crops were maintained according to normal agricultural practice with respect to irrigation, fertilization and pest management measures (Table SI 1.2). However, PPPs applied before the application of the test substances did not contain any of the co‐formulants or a.s. investigated in this work.

Three different PPPs, containing one or two of the selected co‐formulants were applied to each crop (one plot per PPP) 1 week prior to commercial harvest (August 16, 2019). Although not all uses were authorized, the application rates were consistent with agricultural practice in other crops. The pre‐harvest interval of 1 week was chosen to represent typical close‐to‐harvest treatments in vegetables. Treated plots were separated by untreated control plots of equal size to minimize drift to plots treated with a different PPP.

Samples from treated plots were collected 1 day prior to treatment (day −1), 1 h after treatment (day 0), as well as 1, 2, 3, 7, and 14 days after treatment, and additionally on day 21 (only leek). For each sample, eight individual plants per plot were randomly collected, with exception of leek where 12 plants were taken. The samples were brought to the lab within less than 2 h, where non‐edible parts were removed and each plant was cut into halves or quarters in order to reduce the weight to about 2–3 kg per sample. This subsample was cut into pieces (about 5 cm), packed into plastic bags, and stored at −20°C until further processing.

Weather data were available from a meteorological station located only 100 m from the test site. Between day 0 and day 21, daily mean air temperatures were 12 to 23°C. A total of 68 mm of rain was recorded on day 2 to 4 after treatment. Rainfall over the entire sampling period (day 0 to 21) was 132 mm (Table SI 1.3a). No artificial irrigation was necessary after treatment. More details on field trials are reported in SI 1.

An additional field trial was conducted in apples, at a site in 2 km distance from the above test site (47°13′14”N/8°40′02″E, altitude, 550 m, loamy silt soil, pH 7.1). Apple trees (variety: Golden Delicious) were treated with two PPPs containing several of the selected co‐formulants according to normal agricultural practice (Table 1). The application was performed on September 24, 2019, 3 weeks prior to commercial harvest. Samples were randomly collected from 22 individual trees (16 apples per sample directly before treatment (control sample, day −0), 1 h after treatment (day 0), and on days 1, 2, 8, 14 and 21). Further sample processing was as described above. Daily mean air temperatures were 9 to 17°C (day 0 to day 21 after treatment), with 28 mm rainfall after the application (sum of days 0 to 4) and a total of 168 mm over the entire sampling period (Table SI 1.3b).

### 2.2 Sample preparation

Stored samples (−20°C) were further cooled to −45°C over night and then homogenized using a knife mill (Grindomix GM 3000, Retsch). An aliquot of the homogenate (10 g) was processed according to the QuEChERS multimethod for pesticides^12^ using acetonitrile (HPLC gradient grade, VWR) as solvent and QuEChERS tubes I and II (DisQuE 50 mL Tube/AOAC – Acetate and DisQuE 2 mL Tube – AOAC, Waters) for clean‐up. For SDS, DBNS, and docusate extract I (after clean‐up with tube I) was analyzed, and for DMDA, spiroxamine, and trifloxystrobin extract II (after clean‐up with tube I and II). Two replicates of each homogenate were processed and analyzed in separate series on consecutive days. For quality control, fortified samples were processed and analyzed in each series.

### 2.3 LC–MS/MS analysis

Co‐formulants and active substances were analyzed using a LC–MS/MS system consisting of an autosampler (RSI PAL, CTC Analytics, Zwingen, Switzerland), a binary HPLC pump (Sciex Exion LC with microvacuum degasser), and a triple quadrupole mass spectrometer (API 6500^+^, with turbo ion spray source, Sciex Framingham, MA, USA). Analytes were separated by reversed‐phase HPLC on a Gemini‐NX C18 column (5 μm particle size, 150 × 2 mm, protected by a 4 × 2 mm pre‐column with the same stationary phase, Phenomenex, Torrance, CA). Eluents were aqueous ammonium formate (5 mM, puriss p.a., ACS reagent, ACROS ORGANICS, NJ, USA) and methanol (HPLC gradient grade, CHEMSOLUTE Renningen, DE, USA). Gradient elution at a flow rate of 0.2 mL min^–1^ was as follows: initial conditions, 75% methanol, linear increase to 100% within 5 min, followed by 5 min isocratic hold. Initial conditions were then re‐established within 0.1 min and the column equilibrated for 2 min. For more details on LC–MS/MS settings and ion transitions monitored see Table SI 2.1.

### 2.4 Reference materials and quantification

Reference materials were available for DMDA, docusate, SDS, spiroxamine and trifloxystrobin. No reference standard was available for dibutylnaphthalene sulfonate, the lead component of DBNS. We therefore used the technical dispersing agent, a mixture of various (butyl) naphthalene sulfonates. The content of dibutylnaphthalene sulfonate in the dispersing agent was approximately 10%, as estimated from HPLC‐UV analysis assuming identical response of all chromatographically separated components. More details on reference materials are provided in Table SI 2.2

Quantification was based on peak areas compared to external standards prepared in acetonitrile, corrected for the purity of the reference material. No internal standards were used. The calibration ranges were from 0.001 to 1 mg kg^–1^ for docusate and DBNS, 0.001 to 0.5 mg kg^–1^ for DMDA, 0.001 to 0.1 mg kg^–1^ for spiroxamine and trifloxystrobin, and 0.01 to 1 mg kg^–1^ for SDS (Table SI 2.3). Where necessary, samples were diluted to achieve a concentration within the calibration range. For docusate and SDS the respective acids were measured, but residues are expressed as sodium salts. The quantification of spiroxamine was for the sum of its two diastereomers (assuming equal response). DBNS residues were quantified based on the peak area of dibutylnaphthalene sulfonate, but were expressed as the total amount of the dispersing agent. DBNS residues should therefore be considered as semi‐quantitative only, as their precise composition remains unknown.

## 3 RESULTS AND DISCUSSION

### 3.1 Performance of the analytical method

Recoveries were determined for each analyte on several crops at 1–4 fortification levels, measured a few hours after fortification, and were 82–113% for DMDA, 90–105% for docusate, 81–118% for DBNS, 82–110% for spiroxamine, and 97–100% for trifloxystrobin, respectively (range for all crops tested, values are means of all spike levels performed). Recoveries of SDS were lower (57–90%). Therefore, the findings on SDS are associated with a higher level of uncertainty. More details are reported in Table SI 2.5.

The precision of the analytical method was determined based on results from replicate samples and was ±1–25% when expressed as relative deviation of residual concentrations from the mean, with the exception for SDS in leek and few individual other samples, where higher deviations were observed (up to 42%, Table SI 3.1).

Storage stability was tested by comparison of analyte concentrations in extracts freshly prepared from frozen plant material (stored for max. 120 days) to those in extracts prepared from homogenates after storage for an additional 30 to 180 days. In some homogenates segregation of water and organic plant material occurred during storage, leading to an overestimation of residues in plant material. However, when the entire samples were re‐homogenized, reasonable storage stabilities were observed for all matrices: 82–119% (mean over all matrices, 94%) for docusate, 76–130% (108%) for SDS, 77–126% (112%) for DMDA, 94–125% (106%) for DBNS, 72–124% (95%) for spiroxamine, and 76–122% (98%) for trifloxystrobin (Table SI 2.4). Overall, the analytes were considered stable in plant matrices during the relevant storage period.

The limit of quantification (LOQ) was arbitrarily defined as the lowest calibration level (0.001 mg L^–1^ corresponding to a plant residue of 0.001 mg kg^–1^) for all analytes, except for SDS (0.01 mg kg^–1^). At this concentration, signal to noise ratios for the primary mass transitions of all analytes were > 10. Quality control samples showed reasonable recoveries, in the range of 80–120% (mean of two replicates) for most series, but were lower in some series (≥68% when excluding SDS) and higher (148%) for DBNS in parsley, for details see Table SI 3.1. Concentrations in method blanks (distilled water processed in the same way as homogenized samples) were below the LOQ by a factor ≥ 3 for all analytes. Further, concentrations of co‐formulants and a.s. in untreated control samples, collected before treatment, were also low and, in any case, well below those measured after treatment (<0.01 mg kg^–1^ for all analytes in each crop, with the exception of DBNS in leaf lettuce (0.014 mg kg^–1^)).

### 3.2 Formation and decline of co‐formulant residues

All investigated co‐formulants and a.s. were detected in treated vegetable samples that were collected shortly after application of the respective PPPs (day 0‐samples, Table 2). The concentrations were well above the respective LOQs, except in rondini. Overall, the co‐formulant residues were highest for docusate (1.5 to 10 mg kg^–1^, range of residues in vegetables excluding rondini), followed by DMDA (0.3–9 mg kg^–1^), SDS (0.6–6 mg kg^–1^), and DBNS (0.08–0.8 mg kg^–1^). The residues of spiroxamine and trifloxystrobin were in the same range as docusate and DMDA (0.9–11 mg kg^–1^ and 0.8–8 mg kg^–1^, respectively). The distinctly lower residues of DBNS were consistent with the approximately 10× lower application rate, when compared to the other test substances. In rondini, residues of all co‐formulants and a.s. were substantially lower than in the other vegetables (<0.001–0.01 mg kg^–1^), due to the high interception by leaves (see Figs. SI 1.1 and 1.2) and the comparably low surface to weight ratio of the fruits. Therefore, residues in rondini were not considered for further evaluation. In apples residues directly after application were highest for docusate (0.4 mg kg^–1^), followed by SDS (0.2 mg kg^–1^), trifloxystrobin (0.1 mg kg^–1^), and DBNS (0.01 mg kg^–1^). Measured concentrations in individual samples are available in Table SI 3.1.

**Table 2:** Residues of co‐formulants and active substances in vegetables and apples directly after application (day 0) as measured (*C*_*0*_) and after normalisation to an application rate of 1 kg ha^–1^ per substance (*C*_*0,norm*_)

Crop	DMDA (mg kg^–1^)		docusate (mg kg^–1^)		SDS (mg kg^–1^)		DBNS (mg kg^–1^)		spiroxamine (mg kg^–1^)		trifloxystrobin (mg kg^–1^)	
	*C*_*0*_ ^†^	*C*_*0,norm*_ ^‡^	*C*_*0*_ ^†^	*C*_*0,norm*_ ^‡^	*C*_*0*_ ^†^	*C*_*0,norm*_ ^‡^	*C*_*0*_ ^†^	*C*_*0,norm*_ ^‡^	*C*_*0*_ ^†^	*C*_*0,norm*_ ^‡^	*C*_*0*_ ^†^	*C*_*0,norm*_ ^‡^
leek	0.26	*0.52*	1.5	*5.5*	0.6	*3.4*	0.08	*4.0*	0.87	*2.3*	0.80	*3.9*
leaf lettuce	3.2	*6.5*	6.0	*23*	2.2	*12*	0.40	*21*	6.1	*16*	6.0	*29*
head lettuce	5.4	*11*	7.4	*28*	3.5	*19*	0.65	*34*	10.6	*28*	7.5	*37*
parsley	9.0	*18*	10	*39*	6.3	*34*	0.78	*41*	5.3	*14*	6.5	*31*
celery	8.6	*17*	7.3	*28*	3.7	*20*	0.49	*26*	4.8	*13*	4.8	*23*
rondini^§^	<0.001	*n.a*.	0.008	*n.a*.	<0.01	*n.a*.	0.002	*n.a*.	0.004	*n.a*.	0.012	*n.a*.
apples	‐	*‐*	0.40	*1.2*	0.20	*0.8*	0.012	*1.1*	‐	*‐*	0.14	*1.1*

^†^ mean values of 2 separately processed subsamples.

^‡^ application rate in Table 1 was used for calculation of *C*_*0,norm*_.

^§^ measured concentrations were below or close to LOQ and were therefore not normalized.

**Figure 2:**
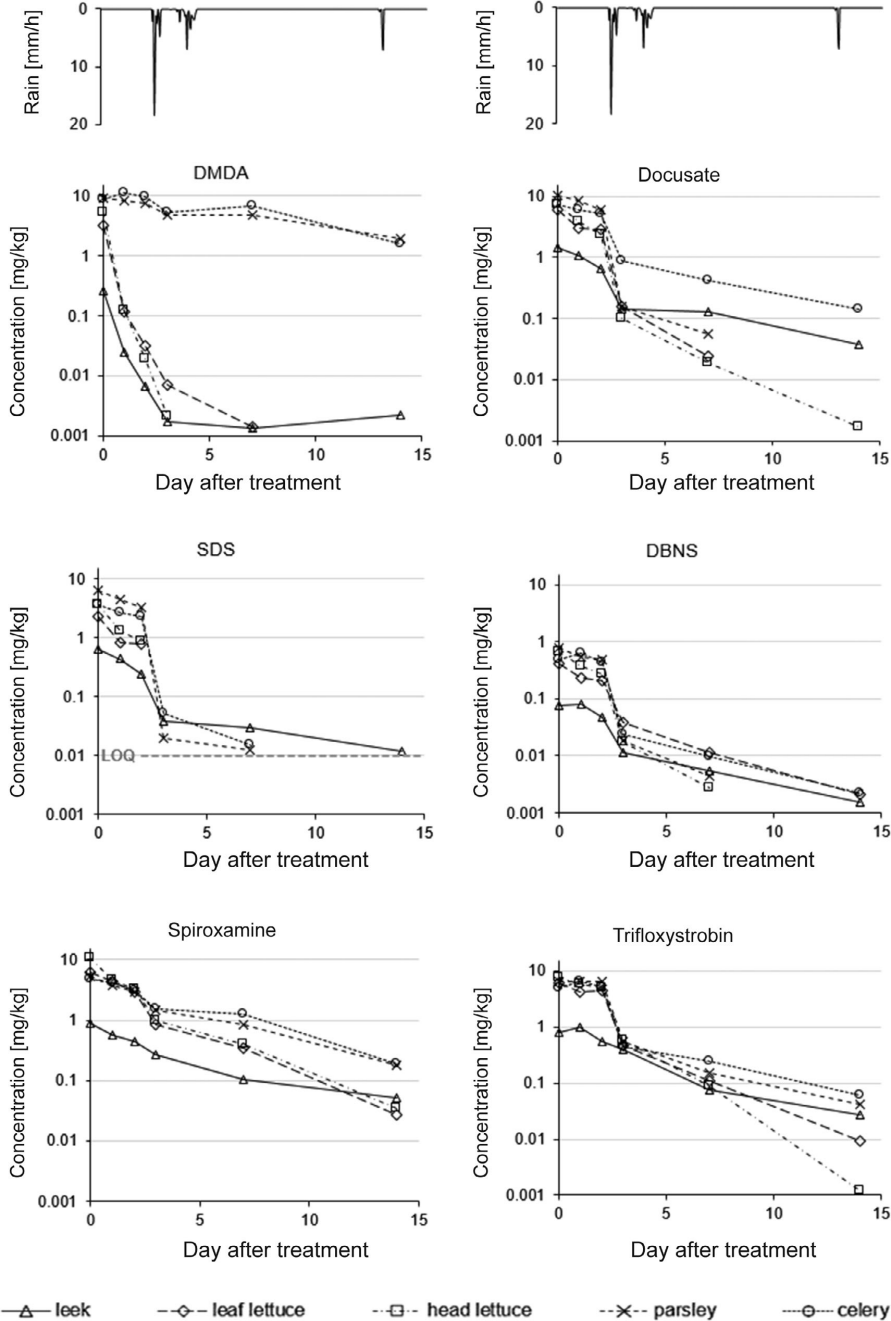
Decline of residues of the co‐formulants DMDA (solvent), docusate, DBNS, SDS (anionic surfactants), and the active substances spiroxamine and trifloxystrobin as measured in five different vegetables. Symbols represent mean values of two replicates, only concentrations >LOQ (both replicates) are shown. LOQ was 0.001 mg kg^–1^ for all substances except for SDS (0.01 mg kg^–1^, as indicated by the grey, dashed line). Top: rainfall intensity (on hourly basis).

The dissipation of the anionic surfactants docusate, SDS, and DBNS from plants (considering leek, lettuce, parsley and celery) was fast, resulting in concentrations of only <1–15% of the initial residues after 3 days. However, in these vegetables the most distinct decline was observed from day 2 to day 3, as illustrated in Fig. 2, concurrently with a rainfall event between samplings on these 2 days (39 mm rain recorded, Table SI 1.3.a). A comparatively less pronounced decline was observed for the a.s. spiroxamine and trifloxystrobin (9–33% and 8–47%, respectively, of the initial residues were measured on day 3). Thus, the faster decline of anionic surfactants in this initial phase indicates that wash‐off is a significant removal process for these co‐formulants, consistent with their high water solubility (>1000× that of spiroxamine or trifloxystrobin, Table SI 1.4). For all anionic surfactants, the decline between day 2 and 3 was least pronounced in leek, possibly because residues were less available for wash‐off in this crop. Two weeks after treatment residues were low for both, anionic surfactants (<3%) and active substances (≤6% of initial concentrations).

The behavior of the organic solvent DMDA differed from that of the anionic surfactants and a.s. Rapid decline to less than 10% of the initial residues within the first day was observed in some of the vegetable crops (leek, leaf lettuce, and head lettuce) and less than 3% and 1% were recovered on day 2 and 3, respectively. This extensive dissipation of DMDA presumably can be attributed to volatilization. Much slower dissipation of DMDA, however, was observed in celery and parsley, with 53% and 62%, respectively, of the initial residues present on day 3, and still 22% and 18% on day 14. Slower decline of residues in celery and parsley may be because DMDA is also quite lipophilic. Strong adsorption of DMDA to the surface of these plants could protect the substance both, from volatilisation and from wash‐off.

**Figure 3:**
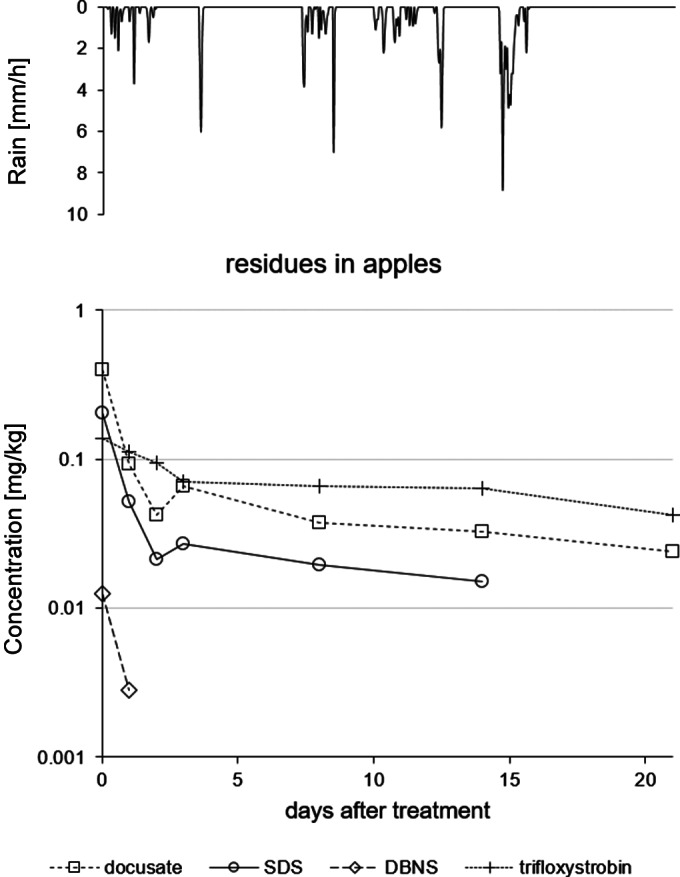
Decline of residues of the co‐formulants docusate, SDS, DBNS (anionic surfactants) and of the active substance trifloxystrobin as measured in apples. Symbols represent means of two replicates; only concentrations >LOQ (both replicates) are shown. Top: rainfall intensity (on hourly basis).

In apples, considerable decline of docusate and SDS residues to roughly 15% of the initial residues was observed within the first 3 days, followed by a much slower dissipation to roughly 10% after 2 weeks. Although frequent rainfall events were recorded between day 0 and 14 (Table SI 1.3b) residues declined slower when compared to vegetables. A direct comparison of the decline curves of the trial in vegetables and in apples is, however, not feasible, as the trials were not conducted during the same time period. Residues of DBNS in apples were low (close to or below LOQ in most samples) and decline is not further discussed. The behavior of trifloxystrobin in apples was qualitatively similar to that of docusate and SDS, with a rapid decline to 52% of the initial concentration until day 3 and much slower decline thereafter to 46% and 31% after 2 and 3 weeks, respectively, indicating again that wash‐off is more important for anionic surfactants than for lipophilic compounds (trifloxystrobin). Decline curves of docusate, SDS and trifloxystrobin in apples are shown in Fig. 3.

### 3.3 Estimation of initial residues

Due to the scarcity of co‐formulant residue data, estimates may provide a useful alternative for the assessment of potential consumer exposure. To predict co‐formulant residues on treated plants, initial concentrations (i.e., determined by deposition of spray on the edible plant parts) need to be estimated in a first step.

Excluding possible losses (e.g., due to volatilization) between application and sampling, the initial residues (day 0‐residues) should primarily be driven by the application rate. Indeed, after scaling of the day 0‐residues to an application rate of 1 kg ha^–1^, these normalized concentrations *C*_*0,norm*_ (Table 2) were in the same range for docusate (5–39 mg kg^–1^), DBNS (4–41 mg kg^–1^), spiroxamine (2–28 mg kg^–1^) and trifloxystrobin (4–37 mg kg^–1^), considering all vegetables (except rondini), and somewhat lower for DMDA (0.5–18 mg kg^–1^).

**Figure 4:**
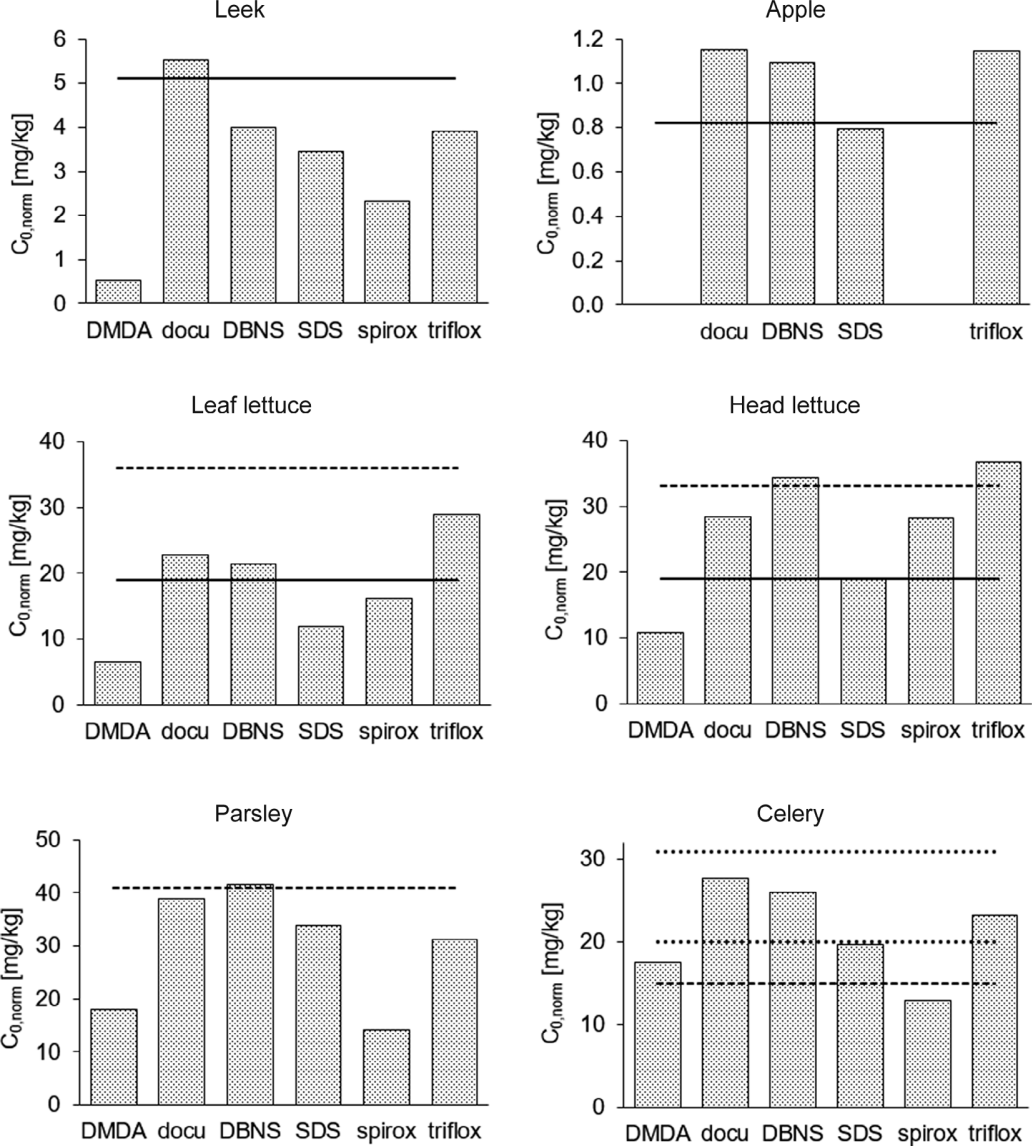
Residues measured directly after application (day 0) and then normalized to an application rate of 1 kg ha^–1^ (*C*_*0,norm*_) for the co‐formulants DMDA, docusate, DBNS, SDS, and the active substances spiroxamine and trifloxystrobin, in comparison to day 0‐residues as reported in Maclachlan and Hamilton.^13^ Leek, leaf and head lettuce, and apple: median values (solid lines). Celery: dotted lines represent the upper and lower 95% confidence interval of the median day 0‐residue (no median value provided^13^). The generic day 0‐residues (*C*_*0,gen*_ *)* calculated with Eq. (1) for leaf and head lettuce, parsley, and celery are also shown (dashed lines). Note the individual scales for each subplot.

Presumably, the initial residues are dependent on factors such as growth stage or planting density, plant architecture and nature of the plant surface, but are expected to be constant for a given crop, when based on a standard application rate. When examined separately for each crop, *C*_*0,norm*_ values for docusate, SDS, and DBNS lie in a very narrow range, that is 3.4–5.5 mg kg^–1^ in leek, 12–22 mg kg^–1^ and 19–34 mg kg^–1^ in leaf and head lettuce, respectively, 34–41 mg kg^–1^ in parsley, 20–28 mg kg^–1^ in celery, and 0.8–1.2 mg kg^–1^ in apples (Table 2 and Fig. 4). Furthermore, these *C*_*0,norm*_ values (as those of spiroxamine and trifloxystrobin) were in good agreement with scaled median day 0‐residues compiled by Maclachlan and Hamilton^13^ from numerous publicly available residue field trials with pesticide a.s. Scaled median values were available for leek, lettuce, and apples (5.1, 19, and 0.8 mg kg^–1^, respectively, Fig. 4). In contrast, measured *C*_*0,norm*_ values of DMDA were lower by a factor of two or more when compared to the other substances in leek (0.5 mg kg^–1^), leaf lettuce (6.5 mg kg^–1^), and head lettuce (11 mg kg^–1^), but within the same range in parsley (18 mg kg^–1^) and celery (17 mg kg^–1^). However, all measured residues were well below the respective 90th percentile of scaled day 0‐residues, that were 6.9, 70, and 2.6 mg kg^–1^ for leek, lettuce and apples, respectively, as reported by Maclachlan and Hamilton.^13^

With a more simplistic approach, generic day 0‐residues (*C*_*0,gen*_ *)* can also be estimated assuming that the crop merely acts as a filter for the spray. Field crops are usually sprayed from above. Their three‐dimensional nature is thus less important than, for example for orchard crops or vineyards. Therefore, the application rate, the portion of the soil surface that is covered by the plants (crop soil coverage), and the plant mass (or crop yield) are the only parameters required to estimate *C*_*0,gen*_ using the following equation:

(eq 1) $$C_{0,\mathrm{gen}}\;\left(\mathrm{mg}\;{\mathrm{kg}}^{-1}\right)=\frac{\text{crop soil coverage}\times \text{application rate}\;\left(\mathrm{kg}\;{\mathrm{ha}}^{-1}\right)\;}{\text{individual plant weight}\;\left(\mathrm{kg}\right)\times \mathrm{No}.\;\text{of plants}\;\left({\mathrm{ha}}^{-1}\right)}\times 10^{6}\;\left(\text{unit conversion}\right)$$

In our field trials we monitored the respective crop parameters for leaf lettuce, head lettuce, parsley, and celery either at the day of application or the day before. The crop soil coverage was determined from photographs by digital image analysis as described by Rasmussen and co‐authors.^14^ For a standard application rate of 1 kg ha^–1^ the resulting estimates for *C*_*0,gen*_ were 36 mg kg^–1^ (leaf lettuce), 33 mg kg^–1^ (head lettuce), 41 mg kg^–1^ (parsley), and 15 mg kg^–1^ (celery), for more details see Table SI 3.2 and Fig. SI 3.1. These values are in good agreement with measured, scaled residues *C*_*0,norm*_ for docusate, SDS, DBNS, spiroxamine, and trifloxystrobin (Fig. 4). However, the above estimate is appropriate only where the consumable commodity more or less comprises the entire plant (i.e., for leafy, but not for fruiting vegetables).

Overall, both scaled median day 0‐residues (as compiled by Maclachlan and Hamilton^13^) and calculation using Eq. 1 provided reasonable (worst case) estimates for day 0‐residues of all tested substances, as demonstrated by comparison with the measured values (Fig. 4). For DMDA, measured values tended to be lower than estimated. This may indicate substantial volatilization during spraying and drying of the spray mixture on the plant surface (compare the relatively high vapor pressure of DMDA, Table SI 1.4).

### 3.4 Estimation of residue decline over time

A first‐tier assessment of consumer exposure for co‐formulants can be conducted solely on the basis of estimated day 0‐residues. However, in some cases a refinement reflecting pre‐harvest intervals may be required. Therefore, in a second step, an estimate for dissipation of a co‐formulant from the edible commodity is needed to predict residues at harvest.

The decline of residues in plants depends on various factors, mainly wash‐off, volatilization, dilution due to plant growth, chemical degradation, and plant metabolism. Hence, decline curves often cannot be described by simple kinetic approaches. Nevertheless, as a starting point within a regulatory framework, it seems justified to apply a simple exponential («single first order», SFO) decline, identified as the (generally) most appropriate kinetic approach by Ebeling and Wang,^15^ and as recommended for pesticide risk assessment for birds and mammals^16^ in the European Union:

(eq 2) $$C_{t}=C_{0}\times e^{-\mathrm{kt}}$$

(eq 3) $$\mathrm{DT}50=\frac{\ln2}{k}$$

where C_0_ is the initial concentration, t the time between treatment and sampling, k the decline rate and DT50 the dissipation half‐life of the substance residues.

Applying SFO fits to the data from our field trials in vegetables, yielded DT50‐values in a narrow range for all crops and similar for all anionic surfactants and a.s. (roughly 1–3 days). Half‐lives of DMDA in leek and lettuce were very short (<0.3 days), but clearly longer in parsley and celery (6 and 7 days, Table SI 3.3a). Although not all decline curves in vegetables were adequately described by SFO, we consider the derived DT50s as indicative.

In apples, decline of docusate, SDS and trifloxystrobin was distinctly bi‐phasic and could adequately be fitted assuming a bi‐phasic kinetic model (Table SI 3.3b). The resulting overall DT50s were 0.4, 0.4, and 5 days, respectively.

The generally short half‐lives that we observed in our field trials are in the same range as those reported in publicly available data reviews on pesticide dissipation on plants. DT50 values for dissipation from foliage of more than 250 active substances from about 400 datasets were evaluated for the EFSA Guidance on Risk assessment for birds and mammals,^16^ originating from work of Willis and McDowell^17^ and the USDA ARS pesticide properties database.^18^ The DT50 values were in the range of 0.1 to 60 days (median, 4 days; 80th percentile, 10 days). In a more recent data compilation, over 2000 data sets of pesticide decline have been evaluated.^19^ When selecting those datasets from crop studies that investigated residues in commodities for human consumption (105 datasets for 48 different a.s.), half‐lives ranged from 0.9 to 69 days (median, 5 days; 80th percentile, 12 days). Another review of 278 residue trials on grass and leafy crops for a selection of 30 currently used a.s. yielded similar median and 90th percentile dissipation half‐lives (3.3 and 7.9 days, respectively).^15^

**Figure 5:**
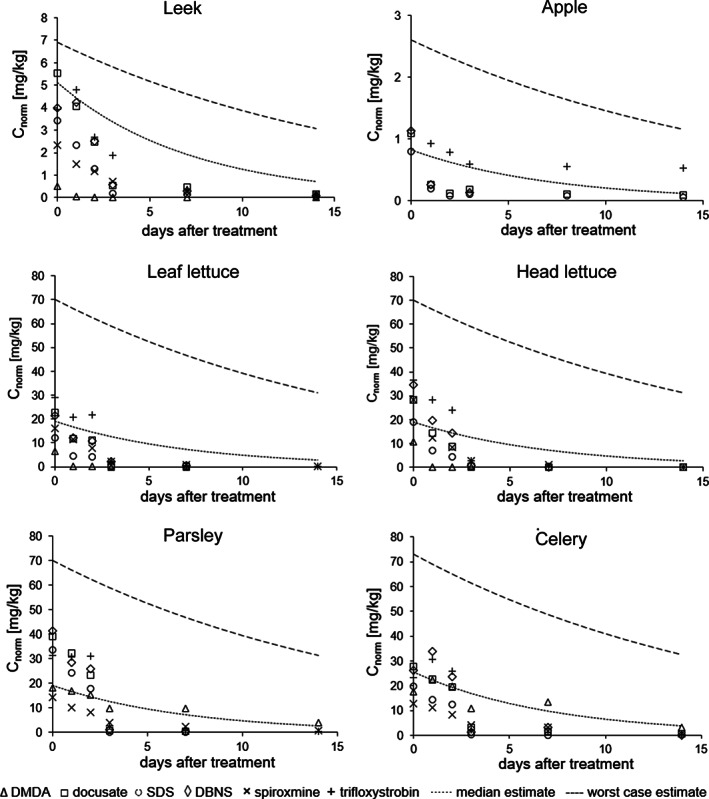
Measured concentrations of co‐formulants and active substances (symbols) after scaling to an application rate of 1 kg ha^–1^ (*C*_*norm*_) in comparison to simulated decline curves. (i) median estimate: SFO decline assuming initial residues based on median day 0‐residue according to Maclachlan and Hamilton^13^ and a median DT50 of 5 d (dotted line). (ii) worst case estimate: SFO decline assuming initial residues based on 90th percentile day 0‐residues^13^ and an 80th percentile DT50 of 12 d (dashed line). Note: for parsley no day 0‐residues were available and values for lettuce were used as surrogate; for celery only few day 0‐values were available, and no median or 90th percentile was provided, we instead applied the mean of the lower and upper median confidence interval and the highest value, as indicated in Maclachlan and Hamilton,^13^ respectively.

We therefore compared the time courses from our field trials (based on measured concentrations scaled to an application rate of 1 kg ha^–1^) of DMDA, docusate, SDS, and DBNS with calculated SFO decline curves based on publicly available data. In a first approach, we assumed median day 0‐residues adopted from Maclachlan and Hamilton^13^ as initial values in combination with the median DT50 of 5 days, as derived from data provided in technical guidance documents.^16^, ^19^ While the simulated concentrations were in the same range as those derived from field trials for the first few days, they generally tended to overestimate actual residues towards the end of the trials, with the exception of DMDA in parsley and celery (dotted lines in Fig. 5). Overall, this approach provided reasonable estimates for the observed magnitude and dissipation of co‐formulant residues.

For regulatory purposes, however, a more conservative assessment of potential consumer exposure might be required. Thus, we also simulated decline curves assuming day 0‐residues based on the 90th percentiles^13^ in combination with the 80th percentile DT50 of 12 days as derived from Lahr *et al*.^19^ The accordingly calculated concentrations consistently overestimated measured residues, providing a clear worst case estimate (dashed lines in Fig. 5). For comparison, we also included measured data of spiroxamine and trifloxystrobin, for which the calculated curves provided reasonable (worst case) estimates as well.

## 4 CONCLUSION

The results of this study build upon a quantitative estimate of co‐formulant contents in PPPs on the market^10^ that allowed for the selection of suitable representative target compounds from two quantitatively important classes (anionic surfactants and solvents). With the sensitive and robust analytical methods developed for the present study, it was possible to determine magnitude and decline of selected co‐formulant residues on edible plant commodities. It was demonstrated, that residues of co‐formulants can occur in considerable amounts (low to medium mg/kg‐range), when analyzed directly after treatment, but these residues, in general, dissipated rapidly with half‐lives of a few days. The decline curves (in combination with weather records) indicated that for anionic surfactants (docusate, SDS, and DBNS) wash‐off was an important removal process. The solvent DMDA exhibited a different behavior, with initial residues that were comparably low, indicating that volatilization may have occurred during application and drying of the spray mixture deposited on the plant surface. Residues of DMDA declined rapidly in lettuce and leek, consistent with the volatility of the compound, but much slower in celery and parsley. Slower decline in the latter crops may suggest that the behavior of lipophilic, volatile co‐formulants needs specific consideration as it may be governed by plant surface properties and not just by volatilization.

For the assessment of actual consumer exposure, reliable information on co‐formulant residues on marketed food, as well as on exposure to the same compounds *via* other routes (e.g., use of everyday products) would be required. However, the number and diversity of co‐formulants in PPPs would make such an assessment very complex. Many co‐formulants are (often not well‐defined) mixtures and, thus, are not easily accessible to analysis at trace levels. For the investigation of (potential) consumer exposure, it therefore will be necessary to estimate co‐formulant residues in food. With our study we demonstrate that it is feasible to predict potential co‐formulant residues based on publicly available data for pesticide active substances. Before applicable in a regulatory framework, the approach would need to be validated including data on other co‐formulant classes (for example, non‐ionic surfactants), crop groups and application patterns and the eventual selection of suitable default parameters (such as initial concentrations and decline rates) will need thorough discussion. Taking into account that up to the present residue data for co‐formulants are not available, our findings provide a key element for future efforts in the assessment of consumer exposure to co‐formulants.

## Supporting information


**Supporting information 1.** Details on field trials, test sites, and selected substances
**Supporting information 2.** Further information on development and performance of the analytical method
**Supporting information 3.** Further information on results and discussion
