# Supplementary material for: Magnitude and decline of pesticide co‐formulant residues in vegetables and fruits: results from field trials compared to estimated values
Source: Pest Manag Sci. 2020 Oct 26;77(3):1187–96. doi: 10.1002/ps.6128 (PMC7894497; doi:10.1002/ps.6128)
Supplement: Supplementary file 1 — Supporting information 1. Details on field trials, test sites, and selected substances Supporting information 2. Further information on development and performance of the analytical method Supporting information 3. Further information on results and discussion [file PS-77-1187-s001.pdf]

Revised 05.10.2020 – Pest Management Science

Magnitude and decline of pesticide co-formulant residues in vegetables and fruits: results from field trials compared to estimated values

Marianne E. Balmer, Daniel Janser, Ulrich Schaller, Jürgen Krauss, H. Christoph Geiser, and Thomas Poiger

Supporting information

Supporting information 1: Details on field trials, test sites, and selected substances

Table SI 1.1: Layout of the test site for the residue trials in vegetables. Dates should be read as dd/mm/yy.

Treatment		crop (variety)					
**PPP** co-formu-lants *a.s.*	application rate (PPP) date	Leek (Belton F1)	rondini (Eight Ball)	celery (Rumba)	parsley (Kisheri F1)	head lettuce (Analotta F1)	oak leaf let-tuce (Katinka)
untreated control 3		3 x 40 plants planting: 03/06/19	15 plants planting: 02/07/19	3 x 18 plants planting: 25/06/19	3 x 18 plants planting: 09/07/19	4 x 18 plants planting: 23/07/19	4 x 18 plants planting: 23/07/19
**Flint** DBNS *trifloxy-strobin*	0.4 kg/ha 16/08/19	3 x 40 plants planting: 03/06/19 BBCH at application: 47-48	15 plants planting: 02/07/19 BBCH at application: 47-48	3 x 18 plants planting: 25/06/19 BBCH at application: 47-48	3 x 18 plants planting: 09/07/19 BBCH at application: 47-48	4 x 18 plants planting: 23/07/19 BBCH at application: 46	4 x 18 plants planting: 23/07/19 BBCH at application: 46
untreated control 2		3 x 40 plants planting: 03/06/19	15 plants planting: 02/07/19	3 x 18 plants planting: 25/06/19	3 x 18 plants planting: 09/07/19	4 x 18 plants planting: 23/07/19	4 x 18 plants planting: 23/07/19
**Input** DMDA *spiroxa-mine; prothio-conazole*	1.25 kg/ha 16/08/19	3 x 40 plants planting: 03/06/19 BBCH at application: 47-48	15 plants planting: 02/07/19 BBCH at application: 47-48	3 x 18 plants planting: 25/06/19 BBCH at application: 47-48	3 x 18 plants planting: 09/07/19 BBCH at application: 47-48	4 x 18 plants planting: 23/07/19 BBCH at application: 46	4 x 18 plants planting: 23/07/19 BBCH at application: 46
untreated control 1		3 x 40 plants planting: 03/06/19	15 plants planting: 02/07/19	3 x 18 plants planting: 25/06/19	3 x 18 plants planting: 09/07/19	4 x 18 plants planting: 23/07/19	4 x 18 plants planting: 23/07/19
**Armicarb** docusate, SDS *potassium hydrogen carbonate ($KHCO_3$)*	3 kg/ha 16/08/19	3 x 40 plants planting: 03/06/19 BBCH at application: 47-48	15 plants planting: 02/07/19 BBCH at application: 47-48	3 x 18 plants planting: 25/06/19 BBCH at application: 47-48	3 x 18 plants planting: 09/07/19 BBCH at application: 47-48	4 x 18 plants planting: 23/07/19 BBCH at application: 46	4 x 18 plants planting: 23/07/19 BBCH at application: 46

plot size: 1.5 m x 7 m.

Seedlings were purchased from Jud Bio-Jungpflanzen AG, Tägerwilen. The application of all plant protection products (PPPs) was conducted with a self-constructed plot sprayer, equipped with IDK 120 04 nozzles (distance between nozzles 25 cm) at a velocity of 3.6 km/h and a pressure of 1.7 bar.

Table SI 1.2: Further information on vegetable test site

Location	Wädenswil, Switzerland (47°22'18''N/8°67'68''E)<br>altitude 485 m above sea level
Soil properties	loamy silt clay 21% (w/w)<br>silt 51%<br>sand 28%<br>pH: 7.1<br>org. C: 4%
Recent field history (cropping)	before 2017 apple orchard<br>2017 pumpkin<br>2018 celery and leaf lettuce
Fertilisation	fertilisation was carried out as follows (dates as dd/mm/yy):<br>24/06/19 celery N: 150 kg/ha P: 40 kg/ha K: 150 kg/ha<br>09/07/19 parsley N: 100 kg/ha P: 40 kg/ha K: 150 kg/ha<br>22/07/19 head lettuce N: 150 kg/ha P: 40 kg/ha K: 150 kg/ha<br>22/07/19 leaf lettuce N: 150 kg/ha P: 40 kg/ha K: 150 kg/ha
Plant protection measures applied in test crops	Prior to treatment with the tested PPPs, products with the following a.s. were applied. These products did not contain any of the investigated co-formulants or a.s.<br>03/07/19 clethodim (herbicide) 120 g/ha leek, rondini, celery<br>30/07/19 azoxystrobin (fungicide) 250 g/ha leaf lettuce, head lettuce<br>08/08/19 pymethrozine (insecticide) 250 g/ha parsley, celery<br>09/08/19 mancozeb (fungicide) 1250 g/ha parsley, celery<br>metalaxyl-M (fungicide) 77 g/ha parsley, celery

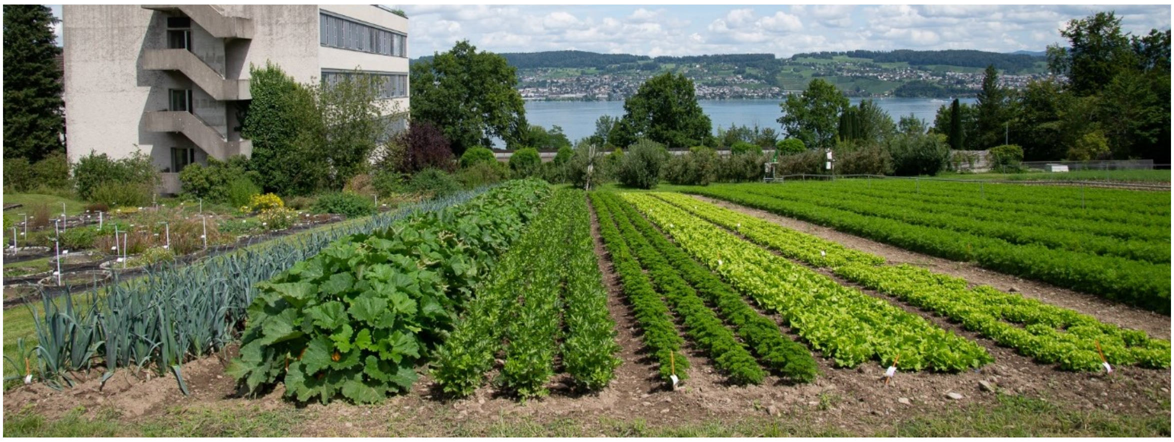

Figure SI 1.1: Vegetable test site in Wädenswil, Switzerland at the day of application (August 16, 2019) with the six tested crops (from left: leek, rondini, celery, parsley, head lettuce and oak leaf lettuce).

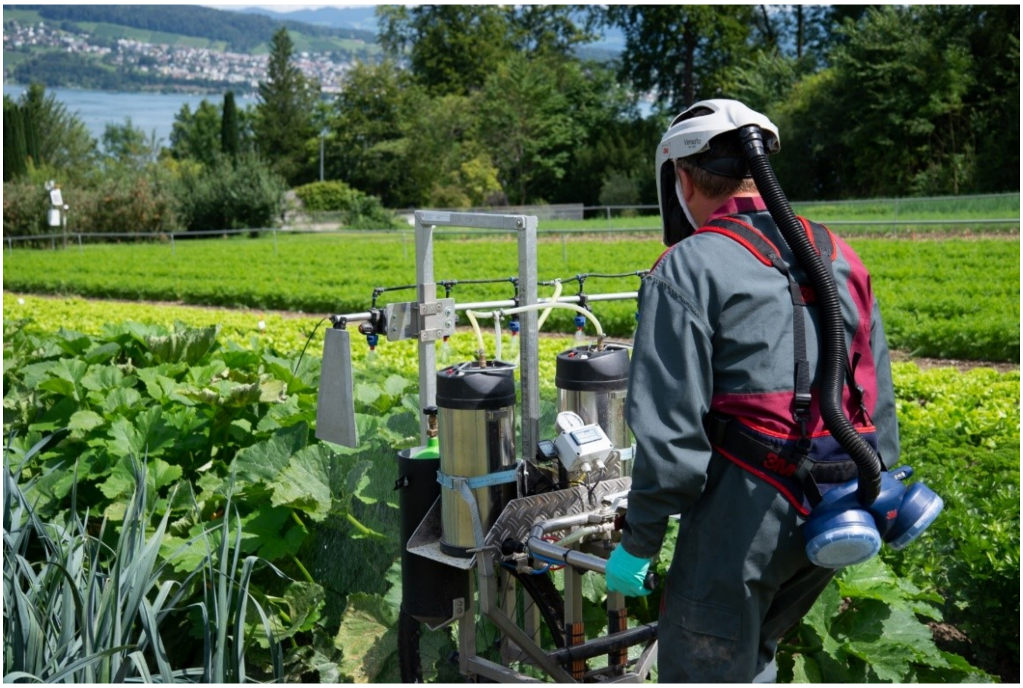

Figure SI 1.2: Application of investigated PPPs at the vegetable test site (crop: rondini).

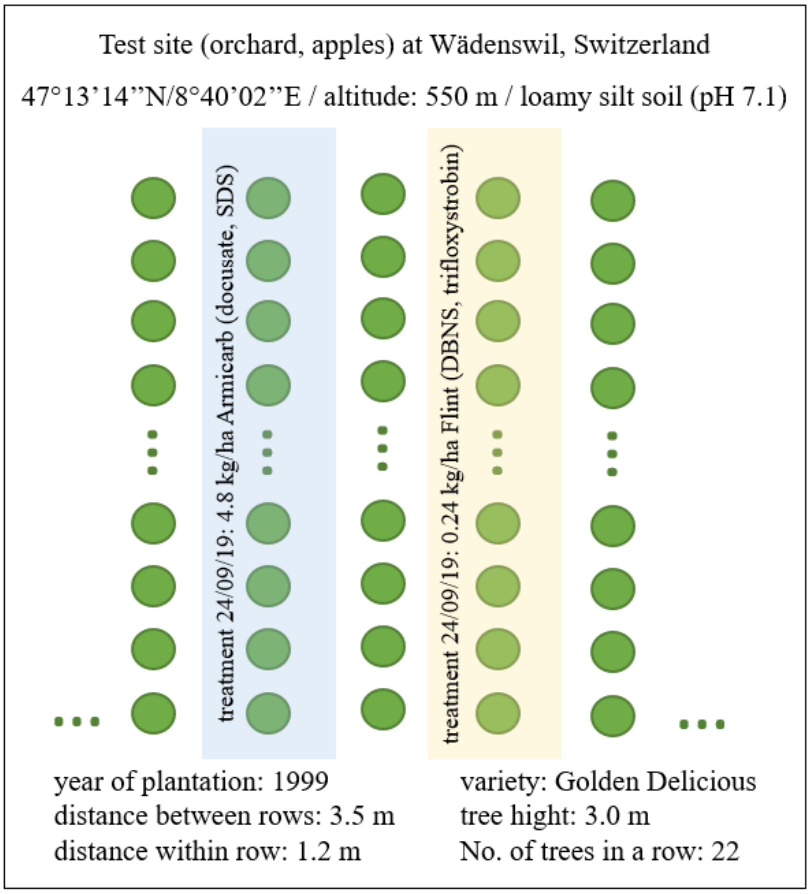

Figure SI 1.3: Layout of the test site for residue trials in apples. In mixture with Flint, an additional PPP containing captan was applied (2 kg/ha of a WG formulation type with 800 g a.s./kg).

Table SI 1.3a: Weather data for the duration of the vegetable field trial sampling period (data from the meteorological station adjacent to the vegetable test site in Wädenswil, Switzerland)

Date (dd.mm.yyyy)	rainfall (mm)	rain intensity (mm/h)	max. temperature (ºC)	min. temperature (ºC)	mean temperature (ºC)	**sampling**
**Vegetable field trial**						
15.08.2019	1.8	6	21.5	11.8	17	**day -1 (control)**
16.08.2019	0	0	24.2	14	18.8	**day 0 (treatment)**
17.08.2019	0	0	25.5	13.8	19.8	**day 1**
18.08.2019	27.8	40.8	31.9	15.8	23.1	**day 2** §
19.08.2019	11.3	15	22.9	16.6	18.9	**day 3** §
20.08.2019	28.9	15.6	16.8	12.7	15.2	
21.08.2019	0	0	20.7	11.9	16.2	
22.08.2019	0	0	17.7	12.6	15.5	
23.08.2019	0	0	20.6	14.7	17	**day 7**
24.08.2019	0	0	25.6	14.3	18.6	
25.08.2019	0	0	29.2	13.8	20.2	
26.08.2019	0	0	29.7	13.3	20.5	
27.08.2019	0	0	29.7	14.3	21.9	
28.08.2019	0	0	29.2	19.3	23.2	
29.08.2019	12.7	25.8	25.2	16.3	20	
30.08.2019	0	0	28.3	15	21.1	**day 14**
31.08.2019	0	0	27.8	16.5	21.1	
01.09.2019	30	35.4	24.1	15.4	18.8	
02.09.2019	9.1	5.4	15.9	11.8	14.6	
03.09.2019	0	0	22.4	10.4	15.7	
04.09.2019	0	0	25.2	11.2	17.6	
05.09.2019	5.4	6	17.8	11.2	13.9	
06.09.2019	6.7	2.4	14.2	10.3	12.2	**day 21**

§ rainfall on day 2 and day 3 was a single rain event. The entire rainfall (a total of 39 mm) occurred between sampling on day 2 and sampling on day 3.

Table SI 1.3b: Weather data for the duration of the apple field trial sampling period (data from the meteorological station adjacent to the vegetable test site in Wädenswil, Switzerland)

Date (dd.mm.yyyy)	rainfall (mm)	rain intensity (mm/h)	max. temperature (ºC)	min. temperature (ºC)	mean temperature (ºC)	**sampling**
**Apple field trial**						
24.09.2019	5.5	7.2	15.3	8.6	12.6	**day -0 (control)**<br>**day 0 (treatment)**
25.09.2019	6.6	14.4	18.8	12.8	14.6	**day 1**
26.09.2019	4	2.4	17.1	12.4	14.5	**day 2**
27.09.2019	2.6	14.4	23.8	13.2	17.1	**day 3**
28.09.2019	8.9	10.2	19.9	11.4	15.4	
29.09.2019	0	0	22.3	9.4	15.9	
30.09.2019	0	0	20.6	10.4	15.9	
01.10.2019	9.7	16.8	21.9	9.8	15.3	
02.10.2019	15.7	22.2	16.1	5.9	12.5	**day 8**
03.10.2019	0	0	14.3	5.4	9.1	
04.10.2019	7.6	3	12.9	6.2	10	
05.10.2019	10.9	3	14.7	10.1	12.1	
06.10.2019	17.6	10.8	15.1	10	11.9	
07.10.2019	0	0	15.7	7.5	11.1	
08.10.2019	0.1	0.6	16.6	7.2	11.8	**day 14**
09.10.2019	56.5	14.4	14.9	8.4	11.5	
10.10.2019	0.2	0.6	16.1	6.7	11.7	
11.10.2019	0	0	17.9	6	10.6	
12.10.2019	0	0	20.2	6.8	13.3	
13.10.2019	0	0	23.5	9.5	14.6	
14.10.2019	0	0	22.1	9.3	14.5	
15.10.2019	21.9	10.8	16	8.8	11.9	**day 21**

Table SI 1.4: Selected properties of the investigated co-formulants and active substances

	substance properties			
	molecular mass [g/mol]	octanol/water partitioning coefficient log Pow [-]	water solubility at 20°C [mg/L]	vapour pressure at 25°C [Pa]
**co-formulant**				
DMDA	199.3 [a]	3.4 [a]	340 [a]	0.11 [a]
docusate	444.6 [a]	[b]	8.2 x 10^{3} [a]	(very low) [c]
SDS	288.4 [a]	[b]	≥ 1.3 x 10^{5} [a]/1 x 10^{5} [d]	(very low) [c]
DBNS	342.4 [a]	[b]	(high) [e]	(very low) [c]
**active substance**				
spiroxamine [f]	297.5	pH 5: 1.28 / 1.41<br>pH 7: 2.79 / 2.98<br>pH 9: 4.88 / 5.08	pH 5: 470 / 340<br>pH 9: 14 / 10	4.0 x 10^{-3} / 5.0 x 10^{-3} (at 20°C)
trifloxystrobin [f]	408.4	4.5	0.61	3.4 x 10^{-6}

Notes to table:

Despite the broad use of many PPP co-formulants in everyday products information on physico-chemical properties is hardly available and/or subject to uncertainties, as e.g. the case for surfactants. In the table we summarize the available information and/or give a qualitative estimate. In contrast, for active substances (a.s.) such information in general is publicly available.

a Substance properties according to the respective registration dossier of the European Chemicals Agency (ECHA), available under https://echa.europa.eu/information-on-chemicals accessed: 20/07/2020

b No reliable log Pow-values are available for surfactants. For docusate ECHA reports a log Pow of 2.0, that was estimated on basis of the water solubility and on the saturation of n-octanol. For SDS a value of ≤ -2.03 is reported by ECHA (see a), estimated in the same way. In contrast, a log Pow for SDS of 1.6 is reported in NLM (reference see d), illustrating the uncertainties coming along with these values.

c Values for vapour pressure were not available for all surfactants. However, based on their chemical structure it can be concluded that docusate, SDS, and DBNS are not volatile (very low vapour pressure). An estimated vapour pressure of 1.6 x 10^{-12} Pa for docusate and a vapour pressure of <1.8 Pa for SDS, respectively is reported by ECHA (reference see a).

d Substance properties according to the data base of the US national library of medicine (NLM), available under https://pubchem.ncbi.nlm.nih.gov/ accessed: 20/07/2020.

e No value for water solubility for DBNS is available. Based on the chemical structure and in comparison with the other surfactants docusate and SDS, a high water solubility can be assumed (grams per litre).

f Substance properties for a.s. are reported according to EFSA's conclusion on spiroxamine[1] and on trifloxystrobin[2], respectively. Separate values are reported for the two diastereomers (cis / trans) of spiroxamine.

[1] European Food Safety Authority; Conclusion on the peer review of the pesticide risk assessment of the active substance spiroxamine. EFSA Journal 2010;8(10)1719. [102 pp.]. doi:10.2903/j.efsa.2010 1719. Available online: www.efsa.europa.eu

[2] EFSA (European Food Safety Authority), 2017. Conclusion on the peer review of the pesticide risk assessment of the active substance trifloxystrobin. EFSA Journal 2017;15(10):4989, 29 pp. https://doi.org/10.2903/j.efsa.2017.4989

Supporting Information 2: Further information on development and performance of the analytical method

Table SI 2.1: Ion transitions and collision energies used for quantification and confirmation of target compounds by LC-MS/MS

	**primary ion transition (*m*/*z*)**	**collision energy (eV)**	**secondary ion transition (*m*/*z*)**	**collision energy (eV)**	**retention time (min)**
positive mode (ion spray voltage, 4.5kV, 250°C)					
DMDA	200.2→102.2	30	200.2→116.1	28	5.5
spiroxamine	298.3→100.1	42	298.3→144.1	27	6.4
trifloxystrobin	409.2→186.0	15	409.2→206.1	10	5.5
negative mode (ion spray voltage, -4.5kV, 250°C)					
docusate	421.2→81.2	-50	421.2→227.1	-30	5.4
SDS	265.1→96.9	-34	265.1→80.0	-90	4.6
DBNS	319.1→289.1	-46	319.1→259.1	-68	3.9

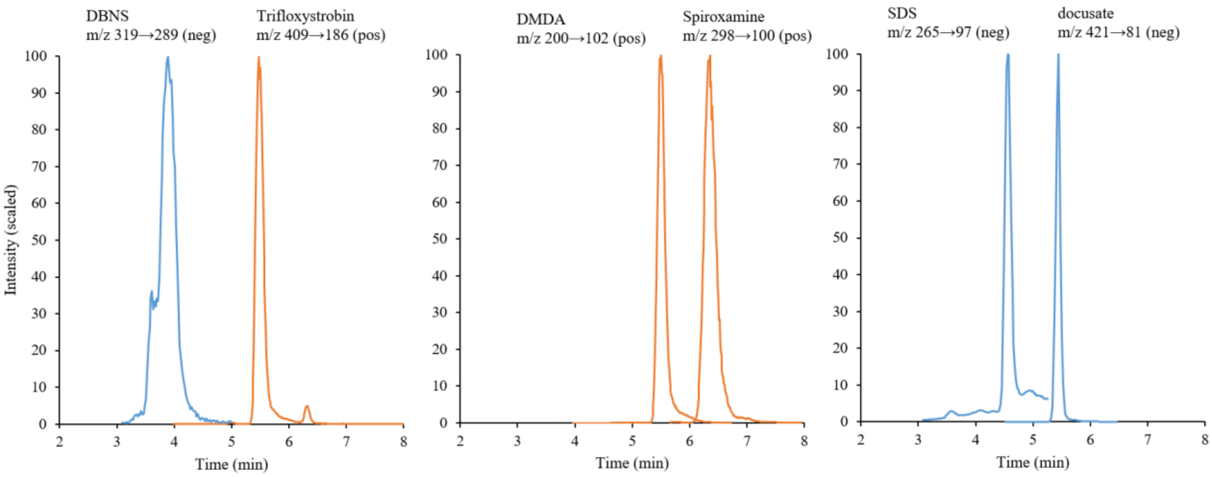

Figure SI 2.1: LC-MS/MS (MRM) chromatograms of extracts of celery (day 3) treated with DBNS and trifloxystrobin (left), DMDA and spiroxamine (middle), or SDS and docusate (right), respectively. The broad peak for DBNS likely is due to the presence of several, partially separated isomers. Likewise, spiroxamine consists of two diastereomers which are only partially resolved under the chosen chromatographic conditions. The small peak at 6.2 min. in the mass trace for trifloxystrobin is a matrix component which did not interfere with our measurements. Note: positive and negative ion transitions were acquired in separate chromatographic runs.

Table SI 2.2: Reference materials used for quantification of analytes

substance		purity	source
DMDA	N,N-dimethyldecanamide	99.1%	Alfa Caesar, Haverhill MA
docusate	dioctyl sulfosuccinate sodium salt	99.6%	ACROS Organics, NJ
SDS	sodium dodecyl sulfate	97.2%	Pan Reac Applichem, Barcelona, Spain
DBNS[a]	dibutyl naphthalene sulfonate	≈10%	TFL Ledertechnik GmbH, Weil am Rhein, Germany
spiroxamine		98.3%	Bayer, Leverkusen, Germany
trifloxystrobin		99.4%	Sigma-Aldrich, St. Louis, MO

[a] Technical product Dispergator B Gran, which consists ≈40-50% of a mixture of various (butyl) naphthalene sulfonates (the reaction product of naphthalene and butanol, sulfonated and neutralised). The content of dibutyl naphthalene sulfonate in the dispersing agent was estimated from HPLC-UV analysis assuming identical response of all individual components. Dispergator B Gran was kindly provided by Syngenta.

Table SI 2.3: Calibration range and concentration levels of reference standards used for quantification of co-formulants and active substances

co-formulant or a.s.	calibration range (mg/kg)	standard series (mg/kg)	correlation coefficient r^2	linear	LOQ (mg/kg)
docusate	0.001 - 1.0	0.001, 0.005, 0.01, 0.05, 0.1, 0.5, 1.0	≥0.99	yes	0.001
SDS	0.01 - 1.0	0.01, 0.05, 0.1, 0.5, 1.0	≥0.98	yes	0.01
DBNS	0.001 - 1.0	0.001, 0.005, 0.01, 0.05, 0.1, 0.5, 1.0	≥0.99	yes	0.001
DMDA	0.001 - 0.50	0.001, 0.005, 0.01, 0.05, 0.1, 0.5	≥0.98	yes	0.001
spiroxamine	0.001 - 0.10	0.001, 0.005, 0.01, 0.05, 0.1	≥0.99	yes	0.001
trifloxystrobin	0.001 - 0.10	0.001, 0.005, 0.01, 0.05, 0.1	≥0.99	yes	0.001

Reference standards were prepared in acetonitrile using the reference substances as given in Table SI 2.2. Standard concentrations were corrected for the purity of the reference substances, except for DNBS, where the quantification refers to the technical product Dispergator B Gran.

Table SI 2.4: Storage stability of co-formulants and a.s., determined by comparison of residues measured in freshly prepared extracts with extracts from the homogenate of the same sample after storage at -20°C during 60-120 days.

**matrix**	**docus-ate** stability (%)		**SDS** stability (%)		**DBNS** stability (%)		**DMDA** stability (%)		**spirox-amine** stability (%)		**trifloxy-strobin** stability (%)	
leaf lettuce	102	#	116	#	109	##	121	#	124	#	76	##
head lettuce	100		115		94		126		75		105	
celery	89		104		102		120		95		122	
parsley	119		130		125		108	##	105	##	127	#
rondini	86	##	*n.a.*		98		120		99		97	#
leek	82		76		96		77		72		66	#
apple	96		106		121		*n.a.*		*n.a.*		94	
mean	*94*		*108*		*106*		*112*		*95*		*98*	

The stability was determined by re-analysis of the samples from day 0, with the exception of some values that were determined from day 1 (#) or day 2 (##) samples. The indicated storage stability values represent the mean as calculated from two separately processed subsamples (value in stored sample expressed in % of the value before storage).

n.a.: not applicable, as residues were below or close to limit of quantification (SDS in rondini) or stability experiments were not conducted (DMDA and spiroxamine in apple).

Table SI 2.5 Determination of recoveries of the analytical methods for the investigated co-formulants and a.s. in vegetables and apple

	docusate	SDS	DBNS	DMDA	spiroxamine	trifloxystrobin
**leaf lettuce**						
spike levels (mg/kg)	0.01; 0.02; 0.1; 0.5	0.05	0.01; 0.05	0.01; 0.02; 0.1; 0.5	0.02; 0.1; 0.5	-
n	5	2	3	5	3	-
recovery (%): mean (range)	105 (93-136)	57 (52-61)	95 (93-101)	97 (87-111)	101 (88-114)	-
**head lettuce**						
spike levels (mg/kg)	0.01; 0.02; 0.1; 0.5	0.05	0.01; 0.05	0.01; 0.02; 0.1; 0.5	0.02; 0.1; 0.5	-
n	5	2	3	5	3	-
recovery (%): mean (range)	95 (90-98)	58 (57-60)	95 (94-96)	98 (91-106)	96 (90-102)	-
**celery**						
spike levels (mg/kg)	0.01; 0.02; 0.1; 0.5	0.05	0.01; 0.05	0.01; 0.02; 0.1; 0.5	0.02; 0.1; 0.5	-
n	5	2	3	5	3	-
recovery (%): mean (range)	95 (88-102)	58 (57-59)	98 (94-100)	96 (84-106)	101 (87-112)	-
**parsley**						
spike levels (mg/kg)	0.01; 0.02; 0.1; 0.5	0.05	0.01; 0.05	0.01; 0.02; 0.1; 0.5	0.02; 0.1; 0.5	-
n	5	2	3	5	3	-
recovery (%): mean (range)	92 (79-109)	68 (67-69)	118 (112-122)	82 (70-89)	82 (74-89)	-
**rondini**						
spike levels (mg/kg)	0.01; 0.05; 0.1	0.05; 0.1	0.01; 0.05; 0.1	0.01; 0.05; 0.1	0.01; 0.05; 0.1	0.005; 0.025; 0.5; 0.1
n	6	4	3	6	6	6
recovery (%): mean (range)	98 (87-121)	90 (82-87)	81 (78-83)	113 (101-129)	110 (89-126)	97 (91-110)
**leek**						
spike levels (mg/kg)	0.01; 0.02; 0.1; 0.5	0.05	0.01; 0.05	0.01; 0.02; 0.1; 0.5	0.02; 0.1; 0.5	-
n	5	2	3	5	3	-
recovery (%): mean (range)	90 (81-102)	67 (62-72)	110 (103-116)	88 (77-95)	88 (78-94)	-
**apple**						
spike levels (mg/kg)	0.02; 0.05; 0.1; 0.5	0.02; 0.05; 0.1; 0.5	0.02; 0.05; 0.1; 0.5	-	-	0.01; 0.05; 0.1
n	6	10	10	-	-	6
recovery (%): mean (range)	94 (74-106)	76 (59-92)	89 (86-110)	-	-	100 (97-104)
overall mean recovery (%)	*96*	*68*	*98*	*96*	*96*	*99*

Supporting Information 3: Further information on results and discussion

Table SI 3.1: Measured concentrations of co-formulants and active substances in vegetables and apples sampled from field trials

Sampling (day)	Replicate		Mean	*deviation from mean [%]*	Replicate		Mean	*deviation from mean [%]*
	A [mg/kg]	B [mg/kg]	[mg/kg]		A [mg/kg]	B [mg/kg]	[mg/kg]	
	**DMDA**							
	**leek**				**apple**			
**QC [%] (spike level)**	90 (0.013)	88 (0.013)	89		-	-	-	-
**-1**	<0.001	<0.001			-	-	-	-
**0**	0.24	0.28	**0.26**	*7.0*	-	-	**-**	-
**1**	0.023	0.026	**0.024**	*7.2*	-	-	**-**	-
**2**	0.008	0.006	**0.007**	*15*	-	-	**-**	-
**3**	0.002	0.002	**0.002**	*5.4*	-	-	**-**	-
**7**	0.001	0.001	**0.001**	*4.0*	-	-	**-**	-
**14**	0.002	0.003	**0.002**	*17*	-	-	**-**	-
**21**	<0.001	<0.001	**<0.001**	-	-	-	**-**	-
	**leaf lettuce**				**rondini**			
**QC [%] (spike level)**	95 (0.23)	91 (0.18)	93		85 (0.061)	78 (0.51)	81	
**-1**	<0.001	<0.001			<0.001	<0.001		
**0**	3.5	3.0	**3.2**	*7.2*	<0.001	<0.001	**-**	-
**1**	0.13	0.11	**0.12**	*8.7*	<0.001	<0.001	**-**	-
**2**	0.028	0.034	**0.031**	*9.2*	<0.001	<0.001	**-**	-
**3**	0.007	0.007	**0.007**	*0.94*	<0.001	<0.001	**-**	-
**7**	0.001	0.001	**0.001**	*2.7*	<0.001	<0.001	**-**	-
**14**	<0.001	<0.001	**-**	-	<0.001	<0.001	-	-
	**head lettuce**				**celery**			
**QC [%] (spike level)**	116 (0.050)	106 0.051)	111		93 (0.13)	84 (0.14)	88	
**-1**	<0.001	<0.001			<0.001	<0.001		
**0**	5.7	5.0	**5.4**	*6.4*	8.8	8.5	**8.6**	*1.3*
**1**	0.18	0.07	**0.12**	*42*	11.9	10.1	**11.0**	*8.1*
**2**	0.023	0.016	**0.020**	*17*	9.8	9.7	**9.7**	*0.49*
**3**	0.002	0.002	**0.002**	*15*	4.9	5.8	**5.3**	*8.7*
**7**	<0.001	<0.001	**-**	-	6.4	7.0	**6.7**	*4.6*
**14**	<0.001	<0.001	**-**	-	1.6	1.5	**1.6**	*0.45*
	**parsley**							
**QC [%] (spike level)**	69 (0.23)	71 (0.23)	70					
**-1**	<0.001	<0.001						
**0**	9.1	8.8	**9.0**	*1.7*				
**1**	8.6	8.0	**8.3**	*3.5*				
**2**	7.3	7.8	**7.5**	*3.0*				
**3**	4.8	4.7	**4.8**	*0.25*				
**7**	4.7	4.8	**4.7**	*0.70*				
**14**	2.0	1.9	**1.9**	*1.5*				

Table SI 3.1: continued

Sampling (day)	Replicate A [mg/kg]	Replicate B [mg/kg]	Mean [mg/kg]	*deviation from mean [%]*	Replicate A [mg/kg]	Replicate B [mg/kg]	Mean [mg/kg]	*deviation from mean [%]*
	**docusate**							
	**leek**				**apple**			
**QC [%] (spike level)**	77 (0.026)	101 (0.026)	89		92 (1.1)	91 (0.12)	91	
**-1**	0.001	0.003			0.001	<0.001		
**0**	1.3	1.6	**1.5**	*7.9*	0.40	0.40	**0.40**	*0.10*
**1**	0.79	1.34	**1.07**	*26*	0.084	0.10	**0.093**	*9.2*
**2**	0.59	0.72	**0.65**	*9.9*	0.045	0.040	**0.042**	*6.2*
**3**	0.12	0.17	**0.14**	*16*	0.072	0.060	**0.066**	*9.1*
**7**	0.11	0.14	**0.13**	*15*	0.038	0.037	**0.037**	*1.3*
**14**	0.032	0.043	**0.038**	*14*	0.035	0.030	**0.032**	*7.2*
**21**	0.011	0.018	**0.014**	*24*	0.022	0.025	**0.024**	*6.1*
	**leaf lettuce**				**rondini**			
**QC [%] (spike level)**	82 (0.13)	80 (0.13)	81		103 (0.027)	103 (0.027)	103	
**-1**	<0.001	<0.001			<0.001	<0.001		
**0**	6.3	5.6	**6.0**	*5.4*	0.007	0.008	**0.008**	*6.4*
**1**	3.2	2.9	**3.1**	*4.8*	0.013	0.013	**0.013**	*1.4*
**2**	3.0	2.8	**2.9**	*3.3*	0.019	0.020	**0.019**	*0.93*
**3**	0.17	0.16	**0.16**	*3.1*	0.003	0.004	**0.003**	*5.9*
**7**	0.025	0.024	**0.025**	*3.7*	0.003	0.003	**0.003**	*1.1*
**14**	<0.001	<0.001	-	-	0.001	0.001	**0.001**	*7.4*
	**head lettuce**				**celery**			
**QC [%] (spike level)**	65 (0.13)	69 (0.13)	67		57 (0.57)	93 (0.57)	75	
**-1**	<0.001	<0.001			<0.001	0.002		
**0**	7.6	7.3	**7.4**	*2.5*	7.0	7.6	**7.3**	*3.9*
**1**	3.6	4.0	**3.8**	*5.6*	5.1	6.8	**6.0**	*15*
**2**	2.3	2.3	**2.3**	*0.47*	4.9	5.2	**5.1**	*3.1*
**3**	0.097	0.108	**0.10**	*5.7*	0.82	0.91	**0.87**	*5.7*
**7**	0.018	0.020	**0.019**	*5.3*	0.40	0.46	**0.43**	*7.2*
**14**	0.002	0.001	**0.002**	*14*	0.13	0.16	**0.14**	*8.7*
	**parsley**							
**QC [%] (spike level)**	121 (0.12)	118 (0.12)	120					
**-1**	<0.001	<0.001						
**0**	10.0	10.5	**10.3**	*2.1*				
**1**	8.6	8.4	**8.5**	*1.3*				
**2**	6.2	6.1	**6.1**	*0.95*				
**3**	0.15	0.16	**0.16**	*2.8*				
**7**	0.054	0.056	**0.055**	*2.2*				
**14**	<0.001	<0.001	-	-				

Table SI 3.1: continued

Sampling (day)	Replicate		Mean	*deviation from mean [%]*	Replicate		Mean	*deviation from mean [%]*
	A [mg/kg]	B [mg/kg]	[mg/kg]		A [mg/kg]	B [mg/kg]	[mg/kg]	
	**SDS**							
	**leek**				**apple**			
**QC [%] (spike level)**	n.a.	n.a.	-	-	83 (1.3)	80 (0.14)	82	
**-1**	<0.01	<0.01			<0.01	<0.01		
**0**	0.53	0.75	**0.64**	*17*	0.20	0.20	**0.20**	*0.21*
**1**	0.30	0.58	**0.44**	*32*	0.047	0.056	**0.051**	*8.4*
**2**	0.21	0.27	**0.24**	*12*	0.022	0.020	**0.021**	*5.0*
**3**	0.026	0.050	**0.038**	*31*	0.028	0.026	**0.027**	*4.8*
**7**	0.022	0.038	**0.030**	*26*	0.021	0.018	**0.020**	*5.4*
**14**	<0.01	0.016	**-**	-	0.017	0.013	**0.015**	*13*
**21**	0.010	0.021	**0.016**	*33*	<0.01	0.010	-	-
	**leaf lettuce**				**rondini**			
**QC [%] (spike level)**	86 (0.15)	75 (0.14)	81		n.a.	n.a.	-	
**-1**	<0.01	<0.01			<0.01	<0.01		
**0**	2.3	2.2	**2.2**	*1.9*	<0.01	<0.01	**-**	-
**1**	0.85	0.79	**0.82**	*3.5*	0.012	0.011	**0.012**	*3.1*
**2**	0.81	0.78	**0.80**	*2.3*	0.013	0.014	**0.013**	*2.9*
**3**	0.015	<0.01	-	-	<0.01	<0.01	**-**	-
**7**	<0.01	<0.01	-	-	<0.01	<0.01	**-**	-
**14**	<0.01	<0.01	-	-	<0.01	<0.01	**-**	-
	**head lettuce**				**celery**			
**QC [%] (spike level)**	65 (0.10)	58 (0.10)	61		102 (0.13)	103 (0.13)	102	
**-1**	<0.01	<0.01			<0.01	<0.01		
**0**	3.6	3.5	**3.5**	*0.4*	3.7	3.6	**3.7**	*1.0*
**1**	1.2	1.4	**1.3**	*6.0*	2.4	2.9	**2.6**	*11*
**2**	0.84	0.85	**0.85**	*0.22*	2.3	2.3	**2.3**	*0.78*
**3**	<0.01	<0.01	-	-	0.050	0.053	**0.052**	*2.9*
**7**	0.011	<0.01	-	-	0.017	0.014	**0.015**	*7.2*
**14**	<0.01	<0.01	-	-	<0.01	<0.01	**-**	-
	**parsley**							
**QC [%] (spike level)**	113 (0.13)	104 (0.13)	109					
**-1**	<0.01	<0.01						
**0**	6.2	6.4	**6.3**	*1.8*				
**1**	4.7	4.3	**4.5**	*3.6*				
**2**	3.2	3.3	**3.3**	*1.8*				
**3**	0.019	0.020	**0.020**	*2.6*				
**7**	0.011	0.014	**0.013**	*11*				
**14**	<0.01	<0.01	-	-				

Table SI 3.1: continued

sampling (day)	replicate A [mg/kg]	replicate B [mg/kg]	mean [mg/kg]	*deviation from mean [%]*	replicate A [mg/kg]	replicate B [mg/kg]	mean [mg/kg]	*deviation from mean [%]*
	**DBNS**							
	**leek**				**apple**			
**QC [%] (spike level)**	89 (0.0093)	94 (0.013)	92		74 (1.2)	74 (1.2)	74	
**-1**	<0.001	<0.001			<0.001	<0.001		
**0**	0.065	0.085	**0.075**	*13*	0.012	0.013	**0.012**	*6.6*
**1**	0.067	0.093	**0.080**	*17*	0.003	0.002	**0.003**	*14*
**2**	0.045	0.049	**0.047**	*3.4*	<0.001	0.0021	-	-
**3**	0.011	0.012	**0.011**	*5.4*	0.001	0.001	0.001	*4.9*
**7**	0.005	0.006	**0.005**	*11*	<0.001	<0.001	-	-
**14**	0.001	0.002	**0.002**	*4.5*	<0.001	<0.001	-	-
**21**	0.002	<0.001	-	-	<0.001	<0.001	-	-
	**leaf lettuce**				**rondini**			
**QC [%] (spike level)**	66 (0.11)	71 (0.11)	69		106 (1.1)	95 (0.12)	100	
**-1**	0.014	0.013			<0.001	<0.001		
**0**	0.41	0.40	**0.40**	*0.70*	0.002	0.002	**0.002**	*12*
**1**	0.22	0.24	**0.23**	*2.5*	0.001	0.001	**0.001**	*1.0*
**2**	0.21	0.20	**0.21**	*0.57*	0.001	0.001	**0.001**	*7.4*
**3**	0.039	0.039	**0.039**	*0.69*	<0.001	<0.001	-	-
**7**	0.011	0.012	**0.011**	*2.1*	<0.001	<0.001	-	-
**14**	0.002	0.002	**0.002**	*20*	<0.001	<0.001	-	-
	**head lettuce**				**celery**			
**QC [%] (spike level)**	104 (0.10)	98 (0.017)	101		110 (0.12)	110 0.11	110	
**-1**	0.008	0.007			0.007	0.006		
**0**	0.66	0.64	**0.65**	*1.5*	0.50	0.48	**0.49**	*2.8*
**1**	0.41	0.33	**0.37**	*11*	0.72	0.55	**0.63**	*13*
**2**	0.27	0.27	**0.27**	*0.26*	0.48	0.41	**0.44**	*7.1*
**3**	0.018	0.016	**0.017**	*7.5*	0.024	0.023	**0.023**	*3.5*
**7**	0.003	0.003	**0.003**	*6.0*	0.009	0.011	**0.010**	*13*
**14**	<0.001	<0.001	-	-	0.002	0.002	**0.002**	*8.4*
	**parsley**							
**QC [%] (spike level)**	146 (0.12)	149 (0.12)	148					
**-1**	<0.001	<0.001						
**0**	0.81	0.75	**0.78**	*3.6*				
**1**	0.53	0.54	**0.53**	*0.68*				
**2**	0.46	0.50	**0.48**	*3.8*				
**3**	0.017	0.020	**0.019**	*7.8*				
**7**	0.005	0.004	**0.004**	*9.7*				
**14**	<0.001	0.002	-	-				

Table SI 3.1: continued

sampling (day)	replicate A [mg/kg]	replicate B [mg/kg]	mean [mg/kg]	*deviation from mean [%]*	replicate A [mg/kg]	replicate B [mg/kg]	mean [mg/kg]	*deviation from mean [%]*
	**spiroxamine**							
	**leek**				**apple**			
**QC [%] (spike level)**	86 (0.010)	94 (0.010)	90		-	-	-	-
**-1**	<0.001	<0.001			-	-	-	-
**0**	0.79	0.95	**0.87**	*9.3*	-	-	**-**	-
**1**	0.53	0.61	**0.57**	*7.1*	-	-	**-**	-
**2**	0.48	0.41	**0.44**	*7.8*	-	-	-	-
**3**	0.26	0.27	**0.26**	*1.2*	-	-	**-**	-
**7**	0.11	0.099	**0.10**	*4.7*	-	-	-	-
**14**	0.057	0.048	**0.053**	*9.3*	-	-	-	-
**21**	0.014	0.013	**0.014**	*0.50*	-	-	-	-
	**leaf lettuce**				**rondini**			
**QC [%] (spike level)**	80 (0.27)	72 (0.26)	76		83 (0.035)	88 (0.26)	85	
**-1**	<0.001	<0.001			0.004	0.001		
**0**	6.3	5.9	**6.1**	*3.5*	0.005	0.004	**0.004**	*3.8*
**1**	4.3	4.3	**4.3**	*0.08*	0.008	0.008	**0.008**	*5.5*
**2**	2.8	3.0	**2.9**	*2.3*	0.007	0.008	**0.007**	*5.8*
**3**	0.88	0.81	**0.85**	*4.0*	0.008	0.009	**0.008**	*8.5*
**7**	0.35	0.34	**0.34**	*2.3*	0.017	0.017	**0.017**	*2.0*
**14**	0.026	0.029	**0.027**	*4.7*	0.008	0.008	**0.008**	*4.6*
	**head lettuce**				**celery**			
**QC [%] (spike level)**	89 (0.049)	88 (0.049)	89		130 (0.14)	113 (0.15)	121	
**-1**	<0.001	<0.001			<0.001	0.001		
**0**	11	10	**11**	*3.3*	4.9	4.8	**4.8**	*1.3*
**1**	5.1	4.2	**4.6**	*9.6*	4.6	3.9	**4.2**	*8.7*
**2**	3.4	3.0	**3.2**	*5.2*	3.0	3.1	**3.1**	*2.5*
**3**	1.3	0.63	**0.99**	*36*	1.4	1.7	**1.6**	*9.0*
**7**	0.50	0.31	**0.41**	*23*	1.2	1.3	**1.2**	*3.4*
**14**	0.037	0.033	**0.035**	*7.0*	0.20	0.18	**0.19**	*3.3*
	**parsley**							
**QC [%] (spike level)**	103 (0.16)	101 (0.16)	102					
**-1**	<0.001	0.001						
**0**	5.4	5.3	**5.3**	*1.3*				
**1**	3.9	3.7	**3.8**	*2.0*				
**2**	2.9	3.1	**3.0**	*3.2*				
**3**	1.5	1.4	**1.5**	*1.1*				
**7**	0.81	0.84	**0.83**	*2.0*				
**14**	0.20	0.17	**0.18**	*7.3*				

Table SI 3.1: continued

sampling (day)	replicate		mean	*deviation from mean [%]*	replicate		mean	*deviation from mean [%]*
	A [mg/kg]	B [mg/kg]	[mg/kg]		A [mg/kg]	B [mg/kg]	[mg/kg]	
	**trifloxystrobin**							
	**leek**				**apple**			
**QC [%] (spike level)**	118 (0.0072)	91 (0.015)	105		74 (0.70)	79 (0.70)	76	
**-1**	<0.001	<0.001			<0.001	<0.001		
**0**	0.77	0.84	**0.80**	*4.4*	0.13	0.15	**0.14**	*6.9*
**1**	0.88	1.10	**0.99**	*11*	0.12	0.10	**0.11**	*6.8*
**2**	0.55	0.55	**0.55**	*0.02*	0.093	0.094	**0.094**	*0.20*
**3**	0.38	0.39	**0.39**	*1.4*	0.072	0.070	**0.071**	*1.5*
**7**	0.059	0.090	**0.075**	*21*	0.066	0.066	**0.066**	*0.25*
**14**	0.023	0.032	**0.027**	*17*	0.064	0.064	**0.064**	*0.43*
**21**	0.009	0.014	**0.011**	*21*	0.035	0.050	**0.042**	*17*
	**leaf lettuce**				**rondini**			
**QC [%] (spike level)**	96 (0.10)	96 (0.10)	96		96 (0.66)	102 (0.11)	99	
**-1**	<0.001	<0.001			<0.001	<0.001		
**0**	5.9	6.0	**6.0**	*0.42*	0.013	0.012	**0.012**	*0.74*
**1**	4.3	4.2	**4.3**	*0.45*	0.009	0.012	**0.010**	*13*
**2**	4.5	4.4	**4.5**	*1.0*	0.012	0.012	**0.012**	*0.9*
**3**	0.44	0.49	**0.47**	*6.0*	0.006	0.005	**0.006**	*5.8*
**7**	0.11	0.11	**0.11**	*0.29*	0.002	0.002	**0.002**	*3.0*
**14**	0.009	0.009	**0.009**	*1.0*	0.002	0.002	**0.002**	*1.5*
	**head lettuce**				**celery**			
**QC [%] (spike level)**	91 (0.064)	91 (0.014)	91		111 (0.054)	111 (0.055)	111	
**-1**	<0.001	<0.001			<0.001	<0.001		
**0**	7.5	7.6	**7.5**	*0.70*	4.8	4.8	**4.8**	*0.21*
**1**	5.9	5.8	**5.8**	*0.65*	6.7	5.9	**6.3**	*6.2*
**2**	4.7	5.2	**5.0**	*4.4*	5.4	5.2	**5.3**	*1.3*
**3**	0.59	0.56	**0.57**	*3.1*	0.43	0.44	**0.44**	*1.2*
**7**	0.081	0.094	**0.087**	*7.9*	0.23	0.24	**0.24**	*2.1*
**14**	0.001	0.001	**0.001**	*1.5*	0.057	0.061	**0.059**	*3.5*
	**parsley**							
**QC [%] (spike level)**	79 (0.11)	85 (0.11)	82					
**-1**	<0.001	<0.001						
**0**	6.5	6.4	**6.5**	*1.2*				
**1**	6.2	6.4	**6.3**	*2.1*				
**2**	6.4	6.3	**6.4**	*1.2*				
**3**	0.56	0.54	**0.55**	*1.7*				
**7**	0.16	0.14	**0.15**	*6.1*				
**14**	0.045	0.039	**0.042**	*7.1*				

QC (quality control): Homogenates of samples from untreated plots were fortified, processed and analysed in each series for the control of method performance. The table represents the recoveries in % and the levels of fortification (spike level) in mg/kg in brackets.

Table SI 3.2: Crop parameters used for generic calculation of day 0-residues and resulting $C_{0,gen}$ values (assuming a standard application rate of 1 kg/ha)

crop	no. of plants per hectare	weight of individual plant (g)	total plant mass per ha (kg)	crop soil coverage (-)	application rate (kg/ha)	$C_{0,gen}$ (mg/kg)
leaf lettuce	95238	171	16311	0.580	1	36
head lettuce	95238	237	22546	0.738	1	33
parsley	71429	158	11317	0.469	1	41
celery	71429	688	49143	0.747	1	15

Notes to table:

The number of plants per hectare was calculated from the actual cropped area of a plot, thus excluding the tractor tracks between plots. The weight of an individual plant was determined as mean of the weight of 6 individual plants sampled on day 0 (before treatment), with exception of celery where the weight was determined as mean of 24 plants sampled on the day before treatment (day -1). These two numbers were used to estimate the total plant mass per hectare at the time of treatment.

Crop soil coverage is the portion of soil (referring to the actual cropped area of a plot) that was covered by plants. It was determined from photographs (top view) taken of the crops the day before treatment (day -1). The pictures were analysed with the online tool as described by Rasmussen and co-authors[3]. «IMAGING Crop Response Analyser» (Version 0.4) is available at https://www.imaging-crops.dk. See also pictures in Fig. SI 3.1 below.

Based on these parameters the generic initial residues $C_{0,gen}$ for leaf lettuce, head lettuce, parsley and celery were estimated for a standard application rate of 1 kg/ha, using the formula presented in the main text.

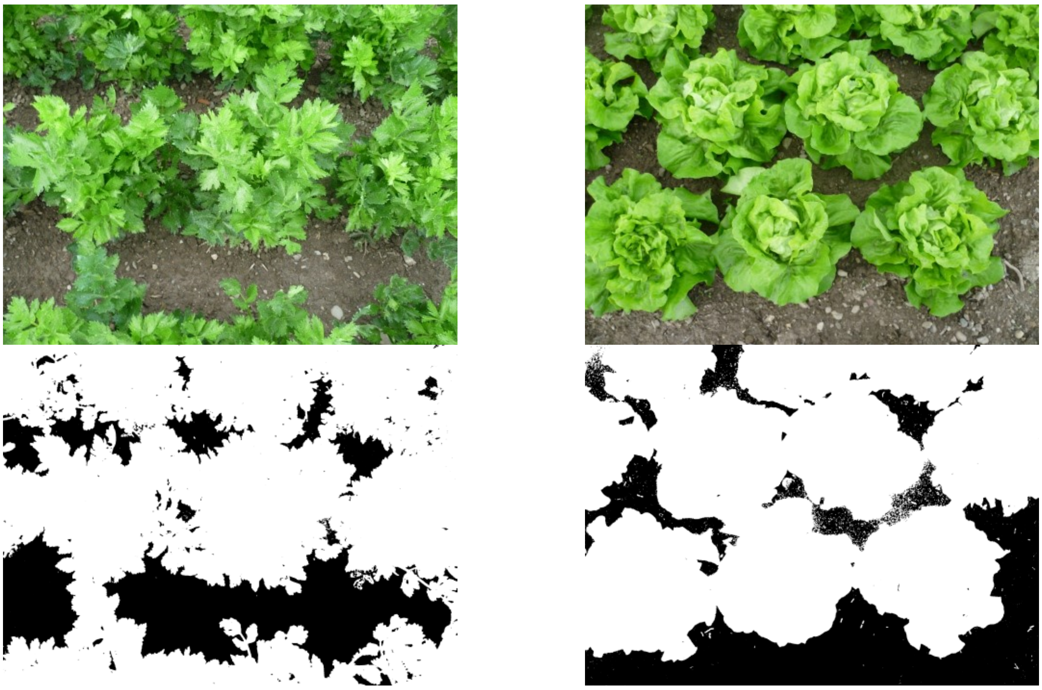

Figure SI 3.1: Photos used for determination of soil coverage and black-white pictures processed by «IMAGING Crop Response Analyser», exemplified for celery (left) and head lettuce (right).

[3] Rasmussen J, Nørremark M and Bibby BM, Assessment of leaf cover and crop soil cover in weed harrowing research using digital images. *Weed Research* **47**:299-310 (2007).

Table SI 3.3a: Results from kinetic fitting of decline over time-curves of co-formulants and active substances in vegetables and apples from field trials, applying SFO (single first order) kinetics

	SFO fit parameter	leek	leaf lettuce	head lettuce	parsley	celery	apple
DMDA	**DT50 (d)**	**0.299**	**0.211**	**0.184**	**6.24**	**7.26**	**-**
	χ^2 (%)	3.84	1.6	0.65	9.85	18.1	
	r^2	0.9997	0.9999	1.0000	0.9076	0.7295	
	visual	acceptable	good	good	acceptable	poor	
docusate	**DT50 (d)**	**1.45**	**1.20**	**0.982**	**1.54**	**1.86**	**0.552**
	χ^2 (%)	16.9	22.2	13.6	27.9	22.9	24.8
	r^2	0.9604	0.9350	0.9800	0.8856	0.8934	0.9748
	visual	acceptable	poor	acceptable	poor	poor	poor
SDS	**DT50 (d)**	**1.25**	n.a.	n.a.	**1.37**	**1.51**	**0.564**
	χ^2 (%)	15.4			24.8	27.7	21.1
	r^2	0.9645			0.9158	0.8871	0.9821
	visual	acceptable			poor	poor	poor
DBNS	**DT50 (d)**	**1.93**	**1.38**	**1.16**	**1.49**	**2.14**	n.a.
	χ^2 (%)	28.1	17.2	17.3	26.7	40.8	
	r^2	0.8830	0.9515	0.9617	0.8939	0.7300	
	visual	poor	acceptable	acceptable	poor	poor	
spiroxamine	**DT50 (d)**	**1.93**	**1.51**	**0.976**	**2.04**	**2.66**	**-**
	χ^2 (%)	7.17	12.4	9.30	8.73	12.2	
	r^2	0.9926	0.9715	0.9892	0.9795	0.9468	
	visual	good	acceptable	good	acceptable	acceptable	
trifloxystrobin	**DT50 (d)**	**2.71**	**1.77**	**1.67**	**2.20**	**2.44**	**12.4**
	χ^2 (%)	23.0	28.1	24.7	36.0	41.2	14.3
	r^2	0.8972	0.8629	0.8978	0.7659	0.6933	0.7510
	visual	acceptable	poor	poor	poor	poor	poor

Notes to table:

For fitting of the decline curves of co-formulants and the two monitored a.s. in vegetables and apples the program CAKE (Version 3.4) was used (available at https://www.tessella.com/showcase/computer-assisted-kinetic-evaluation). The kinetic model has two fitting parameters (initial residue and degradation rate=ln2/DT50); initial residues were not fixed. Residue values <LOQ were set to 0.5 x LOQ for the first time point with a concentration level <LOQ, consecutive time points were not considered then. SFO fits were run for all available co-formulant/crop combinations, provided there where at least five data points available. Examples see Figure SI 3.2.

n.a.: not available (no fits could be run because there were not enough values >LOQ available).

χ^2 (Chi-square) and r^2 are measures for the deviation between observed and predicted values, as described in the respective EU Guidance[4]. Further, the quality of the fits was checked and qualified (good, acceptable, or poor) by visual examination.

[4] FOCUS (2006): «Guidance Document on Estimating Persistence and Degradation Kinetics from Environmental Fate Studies on Pesticides in EU Registration» Report of the FOCUS Work Group on Degradation Kinetics, EC Document Reference Sanco/10058/2005 version 2.0, 434 pp, available at https://esdac.jrc.ec.europa.eu/projects/degradation-kinetics

Table SI 3.3b: Results from kinetic fitting of decline of co-formulants and trifloxystrobin in apples from field trials, applying bi-exponential kinetics (DFOP).

apples DFOP fit	DT50 (days) fast phase	slow phase	g[a]	DT50 (d) overall	DT90 (d) overall	fit quality χ^2 (%)	r^2	visual
docusate	0.0309	16.8	0.859	0.387	8.38	7.66	0.9959	good
SDS	0.350	12.9	0.869	0.429	5.09	6.55	0.9973	good
trifloxystrobin	1.09	32.4	0.460	5.22	78.9	6.17	0.9670	good

[a] g is the fraction of residues at time 0 which is subject to fast degradation

Notes to table:

The decline curves of docusate, SDS, and trifloxystrobin in apples were well described by bi-exponential kinetics (DFOP: double first order in parallel, Figure SI 3.2), assuming a substance is applied to two compartments with fast and slow decline, respectively. Further information and reference, see Notes to table SI 3.3a.

Decline curves of co-formulants and actives in vegetables did not exhibit a clear bi-phasic behaviour. Kinetics other than SFO were, therefore, not applied.

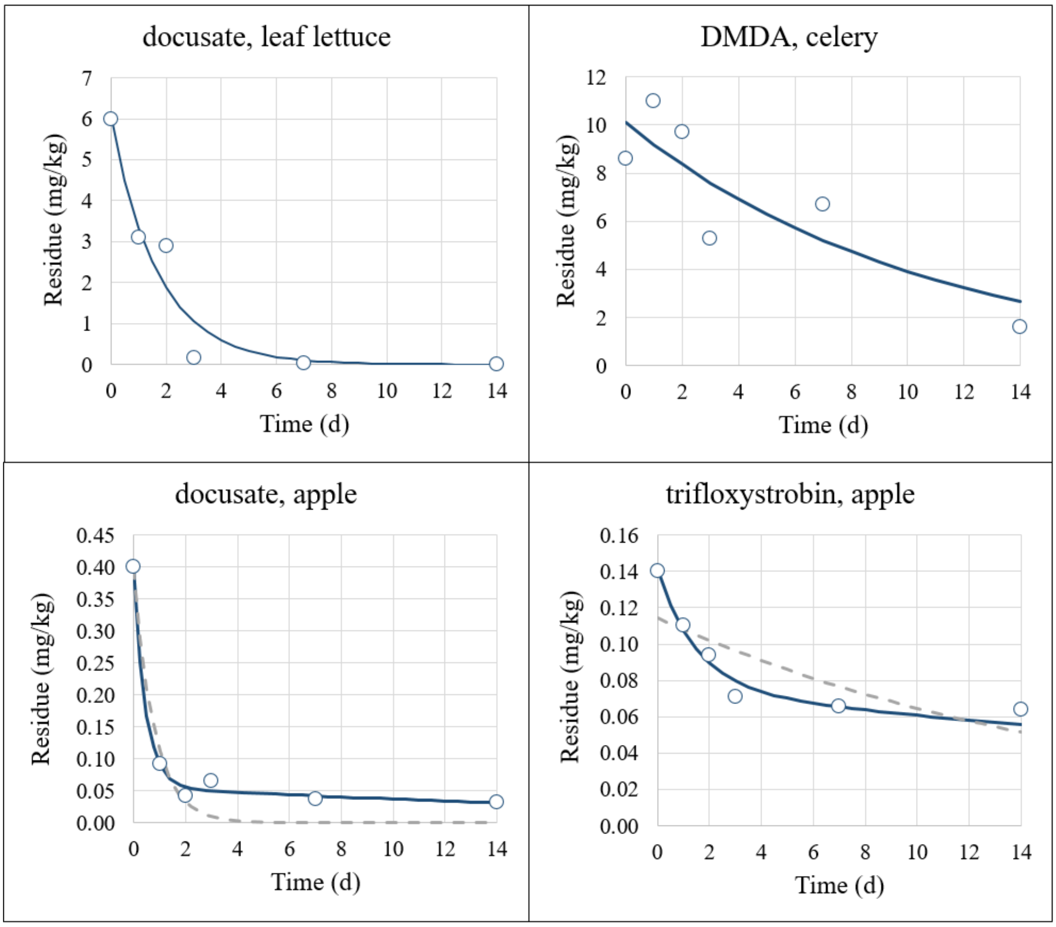

Figure SI 3.2: Example fits of residue data with SFO (top row) and DFOP (bottom row). Dashed lines in bottom panels indicate SFO fits for comparison.
